# Domain architecture and catalysis of the *Staphylococcus aureus* fatty acid kinase

**DOI:** 10.1016/j.jbc.2022.101993

**Published:** 2022-04-29

**Authors:** Chitra Subramanian, Maxime G. Cuypers, Christopher D. Radka, Stephen W. White, Charles O. Rock

**Affiliations:** 1 Department of Infectious Diseases, St. Jude Children’s Research Hospital, Memphis, Tennessee, USA; 2 Department of Structural Biology, St. Jude Children’s Research Hospital, Memphis, Tennessee, USA

**Keywords:** fatty acid, acyl-phosphate, phospholipid, kinase, *Staphylococcus aureus*, ACP, acyl carrier protein, acyl-PO_4_, fatty acyl phosphate, Dha, dihydroxyacetone, FA, fatty acid, FakA, kinase component of fatty acid kinase, FakB, fatty acid binding protein component of fatty acid kinase, SEC-SAXS, size-exclusion chromatography coupled with small angle X-ray scattering

## Abstract

Fatty acid kinase (Fak) is a two-component enzyme that generates acyl-phosphate for phospholipid synthesis. Fak consists of a kinase domain protein (FakA) that phosphorylates a fatty acid enveloped by a fatty acid binding protein (FakB). The structural basis for FakB function has been established, but little is known about FakA. Here, we used limited proteolysis to define three separate FakA domains: the amino terminal FakA_N, the central FakA_L, and the carboxy terminal FakA_C. The isolated domains lack kinase activity, but activity is restored when FakA_N and FakA_L are present individually or connected as FakA_NL. The X-ray structure of the monomeric FakA_N captures the product complex with ADP and two Mg^2+^ ions bound at the nucleotide site. The FakA_L domain encodes the dimerization interface along with conserved catalytic residues Cys240, His282, and His284. AlphaFold analysis of FakA_L predicts the catalytic residues are spatially clustered and pointing away from the dimerization surface. Furthermore, the X-ray structure of FakA_C shows that it consists of two subdomains that are structurally related to FakB. Analytical ultracentrifugation demonstrates that FakA_C binds FakB, and site-directed mutagenesis confirms that a positively charged wedge on FakB meshes with a negatively charged groove on FakA_C. Finally, small angle X-ray scattering analysis is consistent with freely rotating FakA_N and FakA_C domains tethered by flexible linkers to FakA_L. These data reveal specific roles for the three independently folded FakA protein domains in substrate binding and catalysis.

Coenzyme A (1) and acyl carrier protein (ACP) (2) are the two most recognized acyl group carriers in biology. However, in most bacteria, phosphorylated fatty acids (acyl-PO_4_) are the key activated intermediates for the initiation of membrane phospholipid synthesis (3). In the *de novo* pathway, acyl-ACP arising from type II fatty acid synthase is converted to acyl-PO_4_ by PlsX (acyl-ACP:PO_4_ transferase) (Fig. 1). The acyl-PO_4_ is used as an acyl donor by PlsY (*sn*-glycerol-3-phosphate acyltransferase) to synthesize lysophosphatidic acid (3, 4, 5) in the first step in membrane phospholipid synthesis. PlsC uses acyl-ACP to acylate the 2-position to form phosphatidic acid, the key precursor to all phospholipids in *Staphylococcus aureus*. Type II fatty acid synthase is an energy-intensive process, and bacteria also express a fatty acid kinase (Fak) that activates either endogenous fatty acid (FA) arising from phospholipid turnover or exogenous FA for utilization in membrane formation (6, 7, 8). The Fak system consists of two protein components, FakA and FakB, that function together in a cycle (Fig. 1). FakA is the ATP-dependent kinase that phosphorylates a FA bound to FakB, a FA binding protein. The FakBs retrieve the FA from the membrane and deliver it to FakA for phosphorylation. The transfer of the FakA product (acyl-PO_4_ bound to FakB) to lysophosphatidic acid *via* PlsY and to acyl-ACP *via* PlsX has been established and reconstituted *in vitro* (9) (Fig. 1). FakB will also exchange the bound acyl-PO_4_ for a FA present in the membrane (Fig. 1). Bacteria usually express 2 to 6 structurally related FakBs that each optimally bind and transport a specific FA structure that is phosphorylated by a single FakA protein (7, 9, 10). The detailed structural and biochemical analysis of five FakBs has revealed how the protein accommodates the FA within an internal cavity, how the carboxyl group is presented for phosphorylation, and how the protein retrieves FA from the membrane *via* a docking process that involves a structurally encoded conformational change (9, 10, 11, 12). Site-directed mutagenesis identified the residues on FakB that are required for binding the FA and the residues involved in FakB binding to FakA (11). One conserved arginine on FakB is essential for the FakA phosphorylation reaction (Arg173) and a second arginine (Arg205) is key to binding both FakA and the membrane bilayer (11, 12). In contrast to this detailed information about FakB, there is no structural information or mechanistic understanding of the FakA kinase.

**Figure 1:**
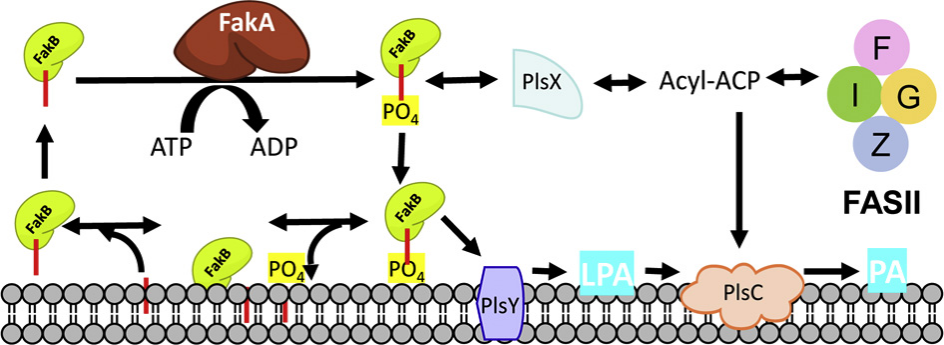
**Role of Fak and acyl-PO**_**4**_**in *S. aureus* lipid metabolism.** Fak is composed of two interacting proteins. FakA is the kinase protein that phosphorylates the FA bound to FakB. FakB is a FA binding protein that picks up FA at the membrane and shuttles them to FakA. Acyl-PO_4_ bound to FakB can exchange with a FA in the membrane if it is present. The reversible PlsX reaction transfers the acyl-PO_4_ from FakB to ACP. Acyl-ACP may be elongated by FASII or be used by PlsC to form phosphatidic acid (PA), the precursor to all phospholipids in *S. aureus*. FakB(acyl-PO_4_) is a substrate for the glycerol-phosphate acyltransferase (PlsY) to form lysophosphatidic acid (LPA) in the first step of phospholipid synthesis. acyl-PO4, fatty acyl phosphate; FA, fatty acid; Fak, fatty acid kinase; FakA, kinase component of fatty acid kinase; FakB, fatty acid binding protein component of fatty acid kinase; FASII, type II fatty acid synthase.

The extensively studied ATP-dependent acetate kinase phosphorylates acetate, a short-chain carboxylic acid (13, 14), is not related to FakA. Instead, different segments of the FakA sequence are related to components of the dihydroxyacetone (Dha) kinase system. Dha kinase is a multiprotein system found in most bacteria that consists of an ADP-binding protein (DhaL), a Dha substrate binding protein (DhaK) and a phosphotransfer protein (DhaM) that phosphorylates the ADP bound to DhaL (15). DhaM phosphorylation is linked to the PEP, HPr, and enzyme 1 phosphotransferase system in bacteria (16). The N-terminal primary sequence of FakA is 24.7% identical to the ADP binding *Escherichia coli* DhaL component, and the carboxy terminus is related to *E. coli* DhaM. The crystal structure of *Citrobacter freundii* Dha kinase is a prototypical example of Dha kinases that exist as single proteins in yeast, animals, plants, and some bacteria (17). In *Citrobacter freundii* Dha kinase, the DhaK piece is lost, the N terminus is related to DhaM/FakB, and the carboxy terminus is related to DhaL (17). Although the C-terminal FakA sequence is related to FakB, FakA neither binds FA nor carries out the Fak reaction in the absence of FakB (7).

The goals of this study are to characterize the FakA structural domains and to define their functions in catalysis. We find FakA consists of three independently folded domains. The N-terminal domain (FakA_N) is the ATP binding component, the central domain (FakA_L) encodes the dimerization function along with a Cys-His-His catalytic triad, and the C-terminal domain (FakA_C) functions to bind the FakB substrate. Small angle X-ray scattering (SAXS) analysis leads to a model where the FakA_N and FakA_C domains are tethered by flexible linkers to the FakA_L dimerization domain. These data identify how each FakA domain contributes to substrate binding and catalysis.

## Results

### FakA has three domains

**Figure 2:**
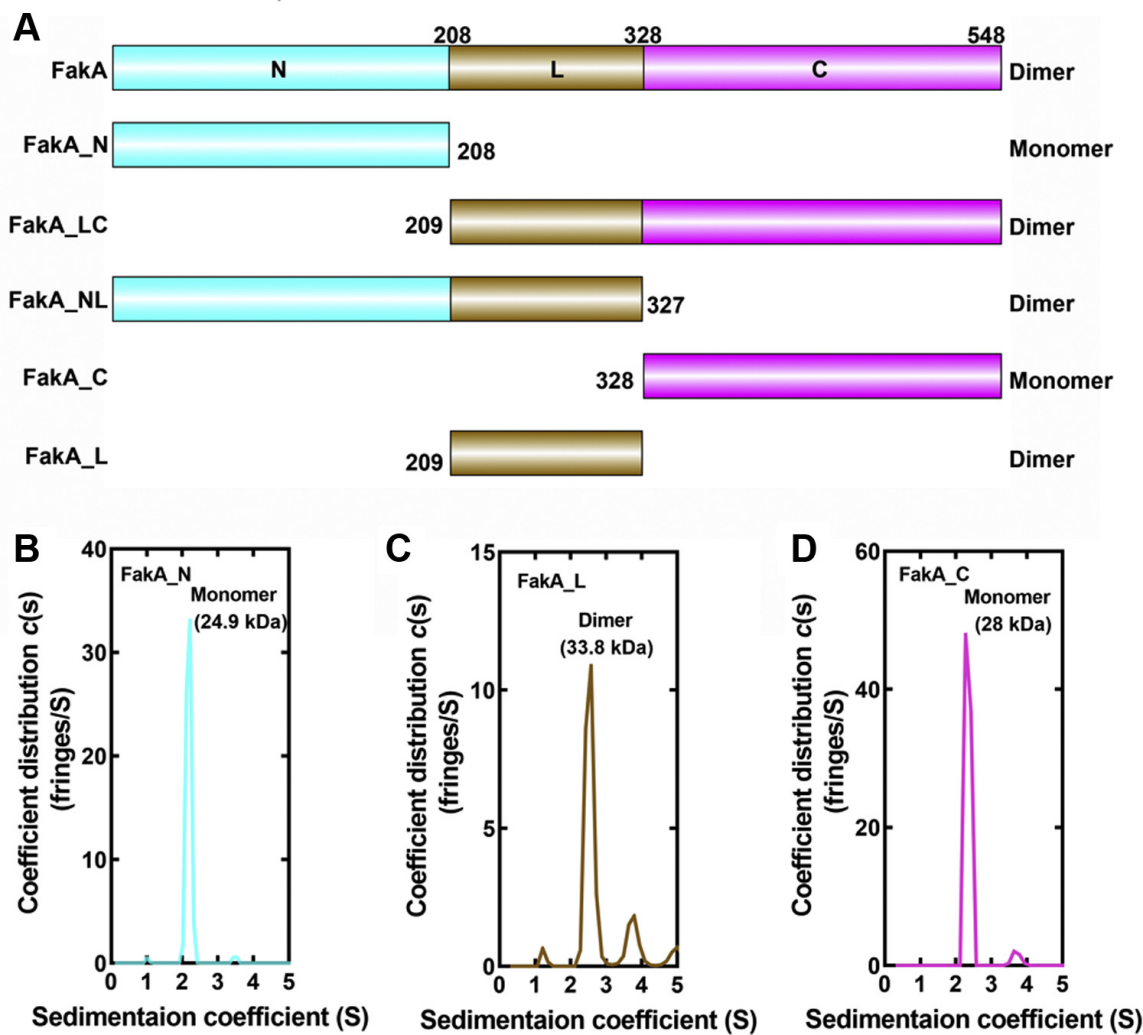
**FakA domain organization.***A*, schematic of FakA domain organization derived from proteolytic digestion. FakA consists of three domains. The FakA N-terminal domain (FakA_N; amino acids 1–208) contains the ATP-binding function. The FakA linker region (FakA_L; amino acids 209–327) encodes the dimerization function and the FakA C terminus (FakA_C; amino acids 328–548) encodes the FakB substrate-binding domain. The oligomerization states of the individually purified protein constructs are listed on the *right*. *B*–*D*, sedimentation velocity profiles (fringe displacement) obtained by analytical ultracentrifugation were fitted to a continuous sedimentation coefficient distribution model c(s) to determine the oligomerization states of the three FakA domains. *B*, FakA_N is a 24.9 kDa monomer. *C*, FakA_L is a 33.8 kDa dimer. *D*, FakA_C is a 28.0 kDa monomer. FakA is a dimer (11). FakA, kinase component of fatty acid kinase; FakB, fatty acid binding protein component of fatty acid kinase.

Purified FakA was subjected to limited trypsin proteolysis that identified two sensitive sites, Lys210 and Lys327. The time for trypsin digestion was empirically optimized, the digestion was scaled to 5 mg/ml FakA, and the products were separated by SDS gel electrophoresis (Fig. S1*A*). The molecular weights of the products indicate two trypsin sensitive sites at residues 210 and 327 that gave rise to five protein products identified by mass spectrometry (Fig. S1*B* and Table S1). These experiments divided FakA into amino terminal (FakA_N), central (FakA_L), and carboxy terminal (FakA_C) domains. Each domain was subcloned, expressed, and purified (Fig. S1*C*). Their solubilities and thermal stabilities reveal that they are stably folded protein domains (Table S3), and their oligomerization states estimated by gel filtration chromatography show they are homogeneous globular entities (Fig. 2*A*). The combined FakA_LC and FakA_NL domains were also cloned, expressed, and purified, and they are both dimers (Fig. 2*A*), which localizes the dimerization domain to FakA_L. Analytical ultracentrifugation confirms the oligomerization state of these three constructs that contain the central domain. FakA_N is a 24.9 kDa monomer (Fig. 2*B*), FakA_L is a 33.8 kDa dimer (Fig. 2*C*), and FakA_C is a 28.0 kDa monomer (Fig. 2*D*). Sedimentation equilibrium statistics for these proteins are found in Table S2. Thermal denaturation experiments showed that FakA and FakA_NL were both stabilized by the presence of ATP•Mg^2+^, whereas FakA_LC was not (Table S3). These data localize the ATP binding function to FakA_N.

### Roles of FakA domains in catalysis

**Figure 3:**
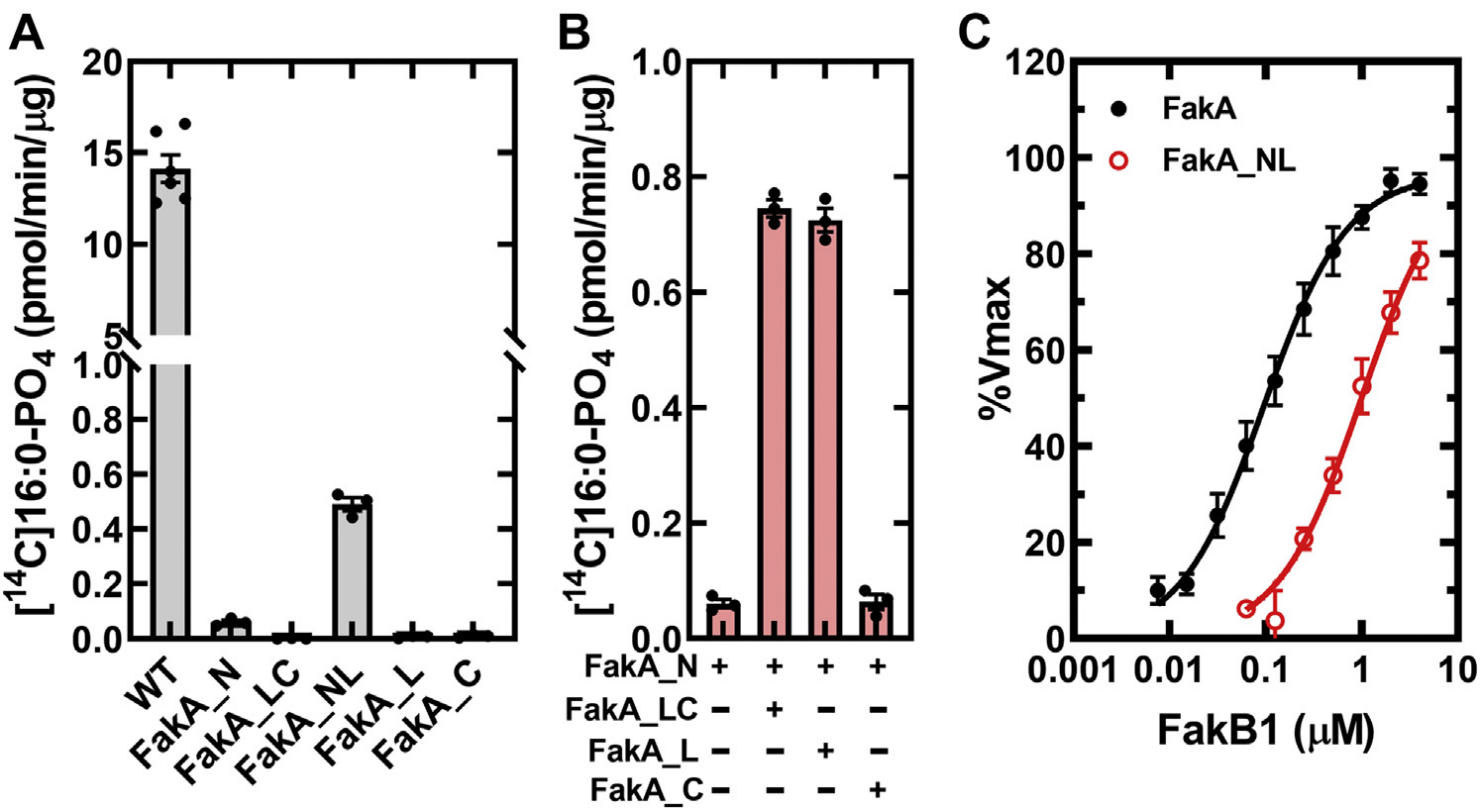
**Reconstitution of Fak activity.** Fak activities of purified FakA domains were measured using the radiochemical kinase assay. *A*, Fak-specific activity compared to the specific activities of the individual FakA domains using 10 μM FakB1 substrate from triplicate experiments. *B*, reconstitution of Fak from inactive FakA domains. FakA_NL (10 μM) was in the linear range (Panel *A*), therefore the activities of the isolated FakA domains (10 μM) were determined in the presence of 10 μM FakA_N in triplicate experiments to determine if FakA_NL activity could be restored by mixing the separate inactive components. *C*, apparent FakB1 K_M_’s for FakA (n = 10) and FakA_NL (n = 4) were 0.09 ± 0.02 μM and 0.99 ± 0.18 μM, respectively (mean ± SE). Fak, fatty acid kinase; FakA, kinase component of fatty acid kinase; FakB, fatty acid binding protein component of fatty acid kinase.

The radiochemical Fak assay was used to determine which FakA domains are catalytically active using FakA as a positive control (Fig. 3). The specific activities of the FakA domains were compared using 10 μM FakB1 to override a potential FakB1 K_M_ defect (Fig. 3*A*). The individual FakA_N, FakA_L, and FakA_C domains are catalytically compromised along with FakA_LC domain fusion (Fig. 3*A*). In contrast, FakA_NL exhibits Fak activity suggesting that residues in both domains are important for catalysis (Fig. 3*A*). In the next series of experiments, FakA_N at 10 μM, the concentration of FakA_NL used in panel A, was incubated with three other protein domains each at 10 μM (Fig. 3*B*). The activity of FakA_N was low, but Fak activity was restored by pairing FakA_N with either FakA_L or FakA_LC, but not with FakA_C. These data support the presence of important catalytic residues within the FakA_L domain. The idea that FakA_NL was defective in FakB1 binding was tested by comparing the apparent FakB1 K_M_ determined with FakA and FakA_NL (Fig. 3*C*). The apparent K_M_ for FakB1 was 0.09 μM for FakA compared to 0.99 μM for FakA_NL. These data show that FakA_NL is a catalytically active Fak, but it has a lower affinity for FakB1.

**Figure 4:**
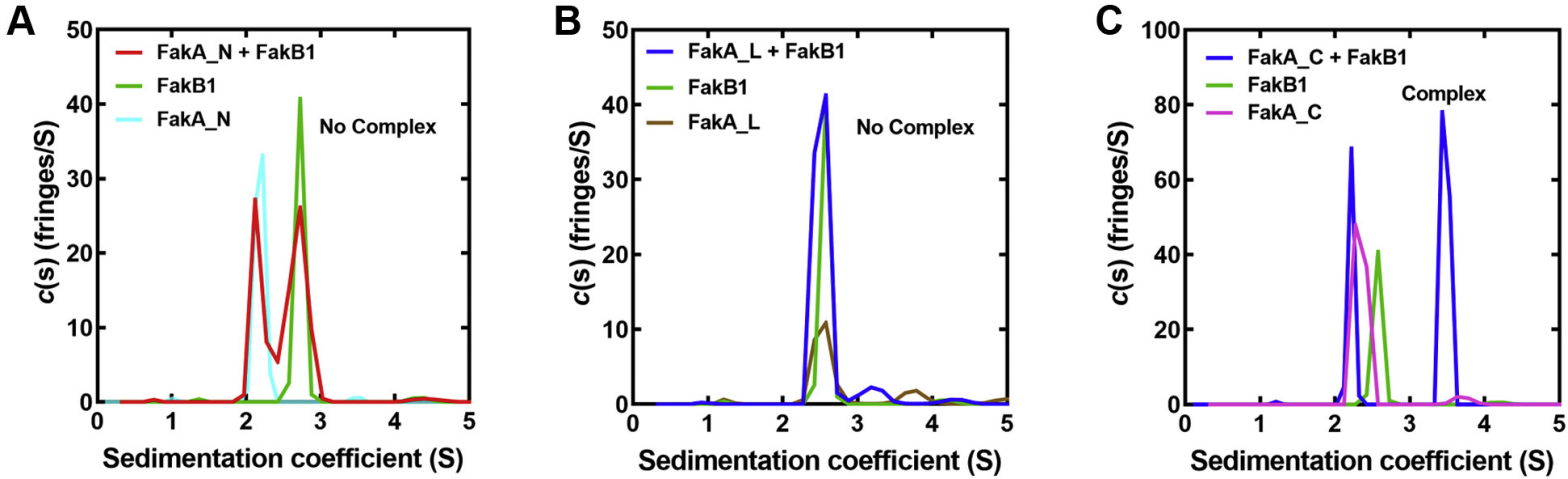
**FakA_C binds FakB.** Sedimentation equilibrium experiments were performed with FakB1 and each of the three isolated FakA domains (Fig. 2). *A*, an interaction between FakB1 and FakA_N was not detected. *B*, an interaction between FakB1 and FakA_L was not detected. *C*, a complex (1:1) of FakB1•FakA_C was detected. FakA, kinase component of fatty acid kinase; FakB, fatty acid binding protein component of fatty acid kinase.

The K_M_ defect in the FakA_NL construct (Fig. 3*C*) suggests that the role of FakA_C is to bind FakB. Analytical ultracentrifugation using the continuous sedimentation coefficient distribution model c(s) was used to test the ability of the three FakA domains to bind FakB1 (Fig. 4 and Table S2). We did not detect FakB1 binding to either FakA_N (Fig. 4*A*) or FakA_L (Fig. 4*B*). However, complex formation is evident when FakB1 and FakA_C are combined (Fig. 4*C*). The molecular weight of the complex is estimated as 62.3 kDa (Table S2), consistent with the binding of one FakB1 (34.5 kDa) to one FakA_C (28 kDa). Based on the equilibrium concentrations measured in our duplicate experiments, the apparent K_D_ for FakA_C binding to FakB1 was estimated as 16 μM using a single site equilibrium model (FakA_C + FakB1 = FakA_C•FakB1). FakB2 binding to FakA has been studied in detail by analytical ultracentrifugation (11), and there are two binding sites that exhibit negative cooperativity with K_D_’s estimated as 1.7 and 6.8 μM. Thus, the affinity of FakA_C for FakB1 appears lower than the affinity of FakA for FakB. These data identify the FakA_C domain as the FakB binding site on FakA.

### Crystal structure of FakA_N

**Figure 5:**
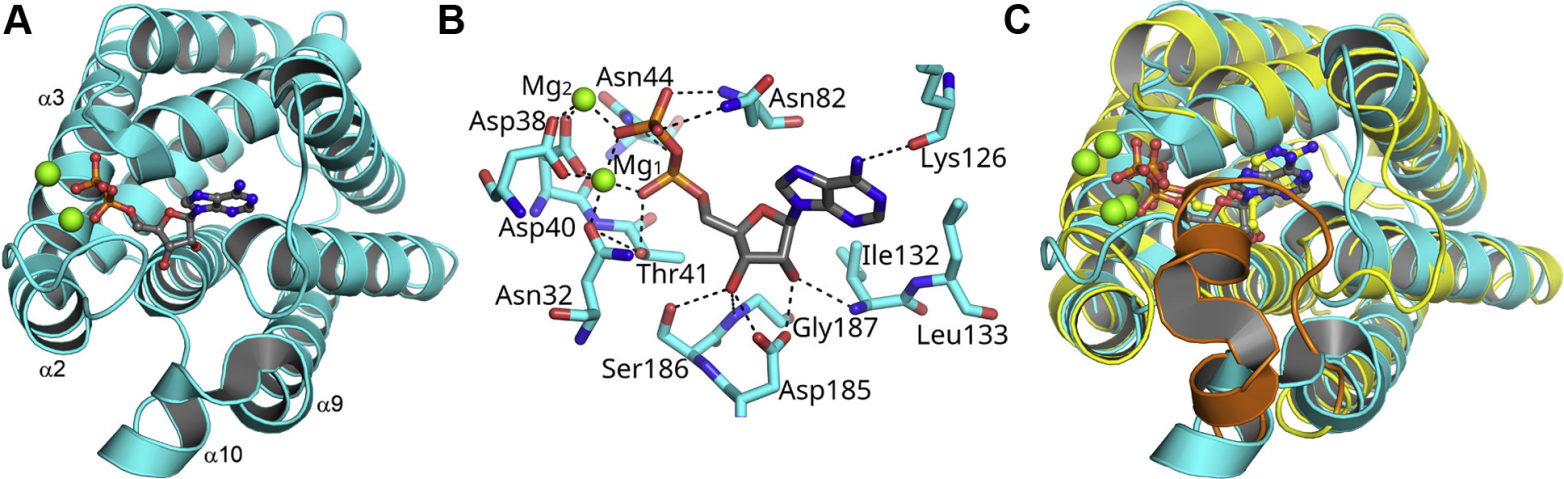
**X-ray structure of FakA_N.***A*, overall structure of FakA_N is formed from 11 α-helices (*cyan*). The bound ADP is shown as *sticks* and the two Mg^2+^ as *green balls*. *B*, the hydrogen bond interaction network surrounding ADP in the FakA_N•ADP•(Mg^2+^)_2_ complex (PDBID: 7RM7). Hydrogen bonds are indicated by *dashed lines*. *C*, overlay of FakA_N (*cyan*) (PDBID: 7RM7) and *E. coli* DhaL (*yellow*) (PDBID: 2BTD) structures. The loop that caps the ADP binding site in DhaL (*orange*) is missing in FakA_N. The DhaL and FakA_N structures have an RMSD deviation of 3.114 Å when the ADP cap of DhaL is excluded from the calculation. RMSD calculations and secondary structure matching was performed using SUPERPOSE/CCP4i.

FakA_N (residues 1–207) produced crystals that diffracted to 1.025 Å, and the structure was determined using a cobalt heavy atom derivative (Table S4). FakA_N expressed in *E. coli* crystallized with a mixture of ADP/AMP and two bound Mg^2+^ ions (Table 1; PDBID: 7SNB). We next crystallized the protein in the presence of adenine nucleotides and Mg^2+^ and solved the structure at 1.025 Å resolution (Table 1; PDBID: 7RM7). These crystals contained ADP and two Mg^2+^ ions. The same ADP-bound structure was obtained regardless of whether ATP or ATP analogs were used in the crystallization trials. The FakA_N structure has a simple architecture comprising 11 α-helices arranged in an up/down barrel configuration (Fig. 5*A*). Located at the end of the helical barrel, the adenine is partially buried in FakA_N by the loop that connects helices α2 and α3 and to a lesser extent by the short helices α9 and α10 (Fig. 5*A*). The key hydrogen bond interactions that fix ADP•(Mg^2+^)_2_ in the binding site include Asp185 that engages the two ribose hydroxyl groups, Asn32, Asp38, and Asp40 that engage the two Mg^2+^ ions, Thr41 that interacts with the α-phosphate of ADP, and Asn82 that interacts with the β-phosphate (Fig. 5*B*).

**Table 1:** X-ray crystallography data collection, refinement, and validation statistics

Protein complex	FakA_N AMP/ADP•(Mg^2+^)_2_	FakA_N ADP•(Mg^2+^)_2_	FakA_C
PDB ID	7SNB	7RM7	6W6B
Data collection			
Beamline	SER-CAT 22-ID	SER-CAT 22-ID	SER-CAT 22-ID
Temperature (K)	100	100	100
Wavelength (Å)	1.000	1.000	1.000
Space group	P212121	P212121	P3121
a, b, c (Å)	42.73, 57.83, 81.23	42.67, 57.40, 81.19	87.30, 87.30, 85.56
α, β, γ	90.00, 90.00, 90.00	90.00, 90.00, 90.00	90.00, 90.00, 120.00
Resolution range (Å)	81.23–1.105	34.24–1.025	75.6–1.41
Rsym or Rmerge	0.061 (0.731)^a^	0.047 (0.743)	0.041 (0.911)
Rpim	0.031 (0.382)	0.020 (0.396)	0.017 (0.386)
Unique reflections	81,058 (4002)	95,789 (3447)	70,140 (3560)
Redundancy	4.8 (4.5)	6.8 (4.3)	6.3(6.4)
Mn I/σ(I)	10.3 (2.0)	19.6 (2.0)	19.1 (2.0)
CC (1/2)	0.997 (0.826)	0.999 (0.650)	0.999 (0.767)
Completeness (%)	99.7 (99.8)	95.5 (70.5)	96.8 (99.5)
Wilson B-factor (Å^−2^)	8.3	8.3	17.8
Model quality			
Rwork/Rfree	15.46/17.07	12.06/14.73	11.9/14.9
No. atoms			
Protein	1720	1831	1846
Ligand/ion	103	55	55
Water	288	362	407
B factor			
All atoms	17.0	18.3	31.6
Protein atoms	15.1	13.8	26.2
Ligands	16.12	17.4	81.0
Solvent atoms	28.68	40.8	49.3
R.m.s. deviations			
Bond lengths (Å)	0.011	0.017	0.021
Bond angles (°)	1.28	2.08	2.12
Protein residues	222	222	233
Ramachandran plot			
Favored (%)	97.7	98.2	98.3
Allowed (%)	2.3	2	1.7
Outliers (%)	0	0	0
Rotamer outliers (%)	0.5	1.5	2.4
Clashscore	2.5	5.6	7.5
Metal ions	Mg	Mg	

a Values in parentheses are for highest-resolution shell.

FakA_N is structurally similar to DhaL, the ADP-binding protein of the Dha kinases (18) suggesting an evolutionary and functional relationship between the two proteins (Fig. 5*C*). The similarity extends to the location and positioning of the nucleotide binding site that is occupied by ADP and two Mg^2+^ in both cases. The main difference between the proteins is an α-helical segment made up of ten amino acids in DhaL that caps the nucleotide binding site (18, 19) and inserts into a cavity on its binding partners DhaK (19) and DhaM (20). This feature is absent in FakA_N (Fig. 5*C*). This difference results in the ADP of DhaL being almost completely buried in DhaL in contrast to FakA_N where the adenine and ribose are enclosed, but the phosphates are exposed to the solvent. The cap region is proposed to limit the rate of ADP exchange in DhaL, which has a half-life of 100 min (16). *Citrobacter* DhaK is similar to FakA_N with the cap replaced by a smaller loop, and this is proposed to allow the observed faster ATP/ADP exchange (17, 18). Another difference is that DhaL has three aspartates (Asp35, Asp35, and Asp37) involved in chelating the two Mg^2+^, whereas in FakA_N, there are only two (Asp38 and Asp40) with the third Mg^2+^ interaction coming from Asn32 (Fig. 5*B*). The FakA double mutant (D38A and D40A) does not complement the Δ*fakA* phenotype (21) confirming that these residues are important for catalysis.

### Catalytic residues in FakA_L

**Figure 6:**
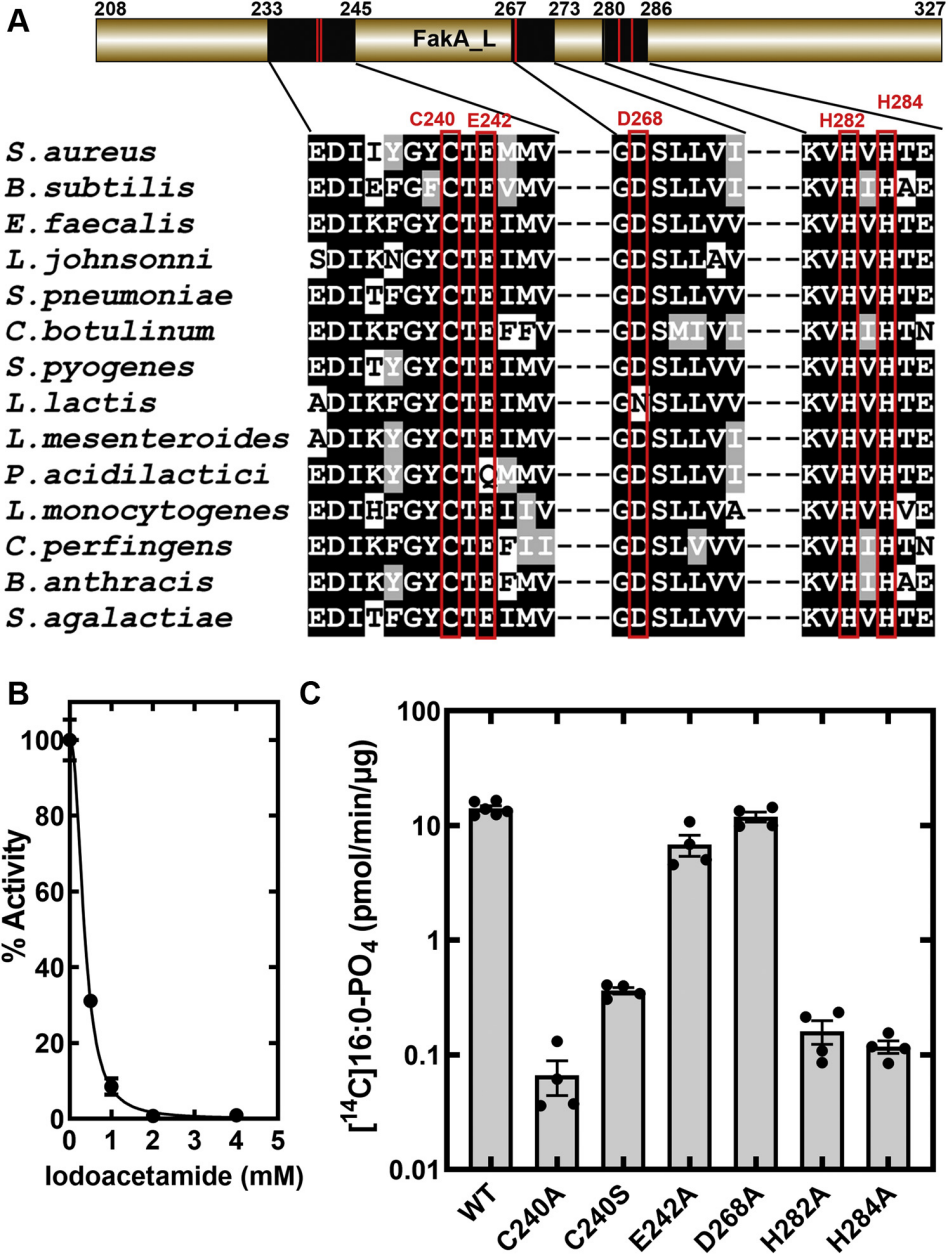
**The role of conserved residues in FakA_L in catalysis.***A*, a schematic diagram of FakA_L showing the location of three blocks of conserved residues in the primary sequence. A pileup of FakA proteins from 14 Firmicute genes illustrates the conserved residues within the FaKA_L domain. *B*, FakA was treated with the indicated concentration of iodoacetamide, and FakA activity was assayed in triplicate. *C*, a log plot of the specific activities of purified FakA mutant proteins. FakA mutants with the indicated residue changes were purified and the specific activities of the FakA mutants were determined in quadruplicate, except for WT where n = 6. FakA, kinase component of fatty acid kinase.

Protein alignment of FakAs from different species showed several highly conserved residues within distinct regions of the FakA_L domain primary sequence (Fig. 6*A*). The first conserved region contains an invariant cysteine (Cys240). Treatment of FakA with iodoacetamide eliminates Fak activity (Fig. 6*B*) indicating the presence of a catalytically important cysteine. Cys240 is the only conserved cysteine among the five cysteines in *S. aureus* FakA. Therefore, we used site-directed mutagenesis to create the FakA(C240A) mutant, which resulted in over two orders of magnitude reduction in FakA-specific activity from 14.14 ± 0.8 pmol/min/μg to 0.067 ± 0.02 pmol/min/μg (Fig. 6*C*). FakA(C240S) also had severely compromised activity but was not as defective as FakA(C240A) (Fig. 6*C*). Catalysis was not affected in the FakA(E242A) and FakA(D268A) mutants (Fig. 6*C*), suggesting that these conserved, but not invariant, residues are not involved in catalysis. There are also two conserved His residues (His282 and His284) arranged in a HΦH configuration (Φ is a hydrophobic residue). Both FakA(H282A) and FakA(H284A) are about 100-fold less active than FakA indicating an important role for the two histidines in catalysis. Gel filtration chromatography and thermal denaturation assays indicate that all mutant proteins are correctly folded and thermally stabilized by ATP•Mg^2+^ (Table S3).

### Predicted structure of FakA_L

**Figure 7:**
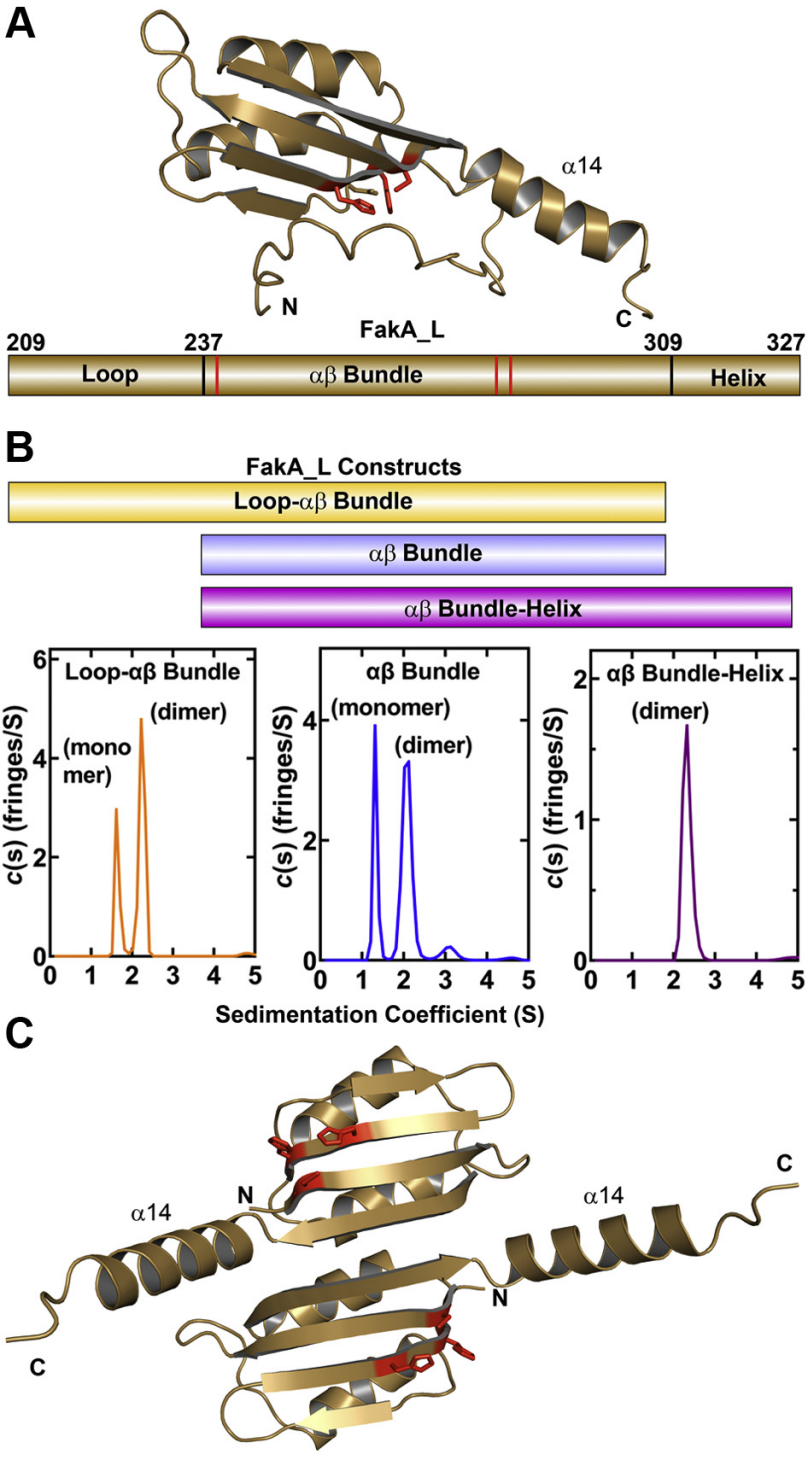
**FakA_L is the dimerization domain.***A*, an AlphaFold analysis of the central domain, FakA_L (*top*), predicts it consists of a core αβ bundle with an unstructured, flexible loop (residues 209–237) connecting it to the N-terminal nucleotide binding domain (FakA_N) (Fig. S3). The C-terminal FakB binding domain is attached by a short linker to helix α14 (residues 309–323) (Fig. S3) that extends from the αβ bundle. The AlphaFold prediction groups the three important catalytic residues in FakA_L together as a Cys-His-His catalytic triad (*red sticks*). Below, a schematic of the FakA_L primary sequence with the three regions labeled and the locations of the Cys240, His282, and His284 indicated by *red bars*. *B*, a schematic of the purified FakA_L constructs and their analysis by analytical ultracentrifugation to determine their dimerization status. Only the αβ bundle–helix formed a tight dimer in solution. *C*, an AlphaFold prediction of the αβ bundle–helix dimer suggest an antiparallel organization of the protomers. FakA, kinase component of fatty acid kinase; FakB, fatty acid binding protein component of fatty acid kinase.

Crystallization trials with FakA_L were not successful, and we therefore used AlphaFold (22) to predict the structure of FakA and its subdomains (Fig. S2*A*). The computed N-terminal domain accurately predicts our FakA_N structure (Fig. S2*B*), and the AlphaFold C-terminal domain matches our FakA_C structure (Fig. S2*C*). AlphaFold predicts that the FakA_L monomer consists of three segments (Fig. 7*A*): an N-terminal unstructured loop, a central αβ Bundle, and a C-terminal helix (α-14). The β-sheets and α-helices are numbered according to the assembled FakA structural alignment with the primary sequence (Fig. S3). The predicted FakA_L structure also places Cys240, His282, and His284 together in a catalytic triad configuration (Fig. 7*A*) consistent with the proposed roles of these residues in catalysis (Fig. 6). Three FakA_L constructs were purified and analyzed by analytical ultracentrifugation to determine which elements of the predicted structure are involved in dimerization (Fig. 7*B*). The loop-αβ bundle and αβ bundle proteins exist in a monomer–dimer equilibrium; however, the αβ bundle–helix construct is a tight dimer (Fig. 7*B*). These data show that the αβ bundle and helix α14 are required for high-affinity dimerization. The dimer structure was predicted with AlphaFold (22) using two αβ bundle-helix sequences concatenated with a flexible 30 residue poly-glycine linker as the input (Fig. 7*C*). This analysis generated an antiparallel assembly in which the αβ bundle from each monomer associates with its dimeric partner *via* the β-strands with the Cys-His-His catalytic triad pointing away from the dimer interface. The α-14 helices are at opposite ends of the antiparallel assembly.

### Crystal structure of FakA_C

**Figure 8:**
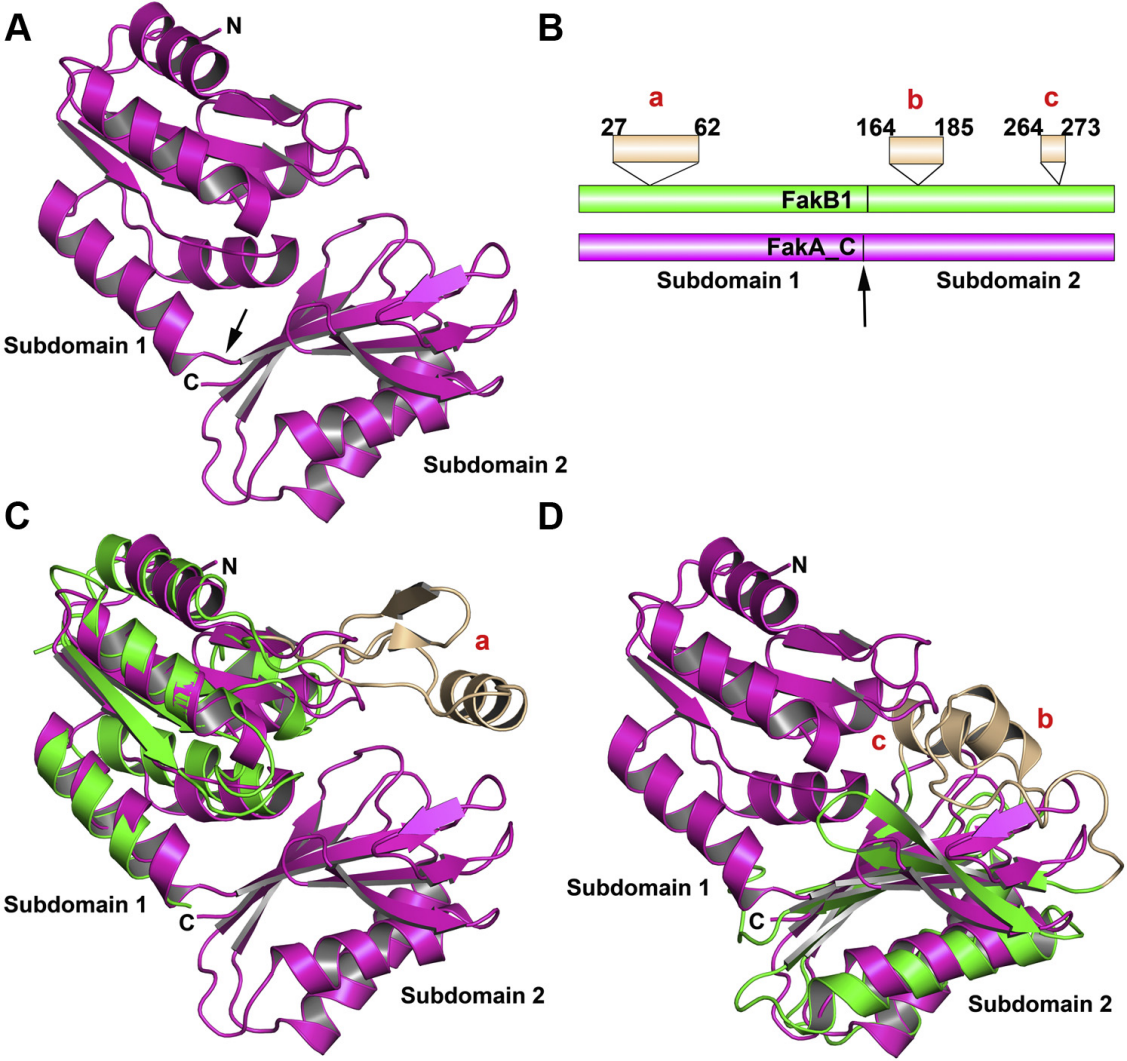
**Crystal structure of FakA_C.***A*, FakA_C consists of two subdomains. The *arrow* indicates where the FakA_C subdomains are connected. *B*, diagram of the structural alignment of FakB1 (*green*) with FakA_C (*magenta*). The three elements of FakB that do not match a structural element in FakA_C (a, b, c) are shown in *tan*. *C*, alignment of the N terminus of FakB1 (*green*; PDBID: 5WOO) and subdomain 1 of FakA_C (PDBID: 6W6B; *magenta*) structures with the nonaligned FakB1 (a; residues 27–62) shown in *tan*. The N terminus of FakB1 and FakA_C subdomain 1 structures have an RMSD of 2.19 Å when the inserted sequence (*tan*) is excluded from the calculation. *D*, overlay of the FakB1 C terminus (*green*; PDBID: 5WOO) with FakA_C subdomain 2 (*magenta*; PDBID: 6W6B) with the two FakB1 segments that do not align for FakA_C shown in *tan* (b, residues 164–185; c, residues 264–273). The C terminus of FakB1 and FakA_C subdomain 2 structures have an RMSD deviation of 3.05 Å when the inserted sequences (*tan*) are excluded from the calculation. RMSD calculations and secondary structure matching was performed using SUPERPOSE/CCP4i. FakA, kinase component of fatty acid kinase; FakB, fatty acid binding protein component of fatty acid kinase.

The 1.40 Å crystal structure of FakA_C was determined using sulfur phasing (Table 1; PDBID: 6W6B). The FakA_C structure consists of two α/β subdomains encompassing residues 328 to 444 and 445 to 548 (numbering with respect to full length FakA; Fig. S3). Subdomain 1 contains a four-stranded parallel β-sheet flanked by two α-helices on one side and three on the other (Fig. 8*A*). Subdomain 2 contains a mixed six-stranded β-sheet flanked by two α-helices on one side and subdomain 1 on the other side (Fig. 8*A*). The two FakA_C subdomains are each related to segments of FakB1. The similarities are difficult to immediately discern because FakB1 has one insertion into the FakA_C subdomain 1 sequence and two insertions into subdomain 2 (Fig. 8*B*). Subdomain 1 of FakA_C corresponds to what is called the EDD domain (23) that is present in the mannose transporter EIIA, DhaK, FakB, and tubulin. Subdomain 1 is structurally related to the N-terminal segment of FakB1 (Fig. 8*C*), but the FakB1 EDD domain differs in having a 35 residue α/β insert that is not present in the canonical EDD structure (Fig. 8, *B* and *C*). Subdomain 2 of FakA_C is structurally related to the FakB1 C-terminal segment (Fig. 8*D*). In this case, there are two insertions in the FakB1 sequence (Fig. 8*B*) compared to the FakA_C structure (Fig. 8*D*). The subdomain 2-fold is related to the FtsZ C terminus (15) (PDBID: 3VO9; residues 225–315; RMSD of 2.868 Å). The structural elements in FakB1 that are missing in FakA_C contribute to the FA binding tunnel that is not present in FakA_C (Fig. S4). The FakB1 tunnel is formed by a crevice at the interface of the N- and C-terminal segments, and the three insertion sequences (Fig. 8*B*) come together in the FakB1 three-dimensional structure to form the top of the FA tunnel (Fig. S4*A*). When these structural elements are missing in FaKA_C, all that remains of the FA binding tunnel is an open crevice on the FakA_C surface (Fig. S4*B*).

### FakA_C binding to FakB1

**Figure 9:**
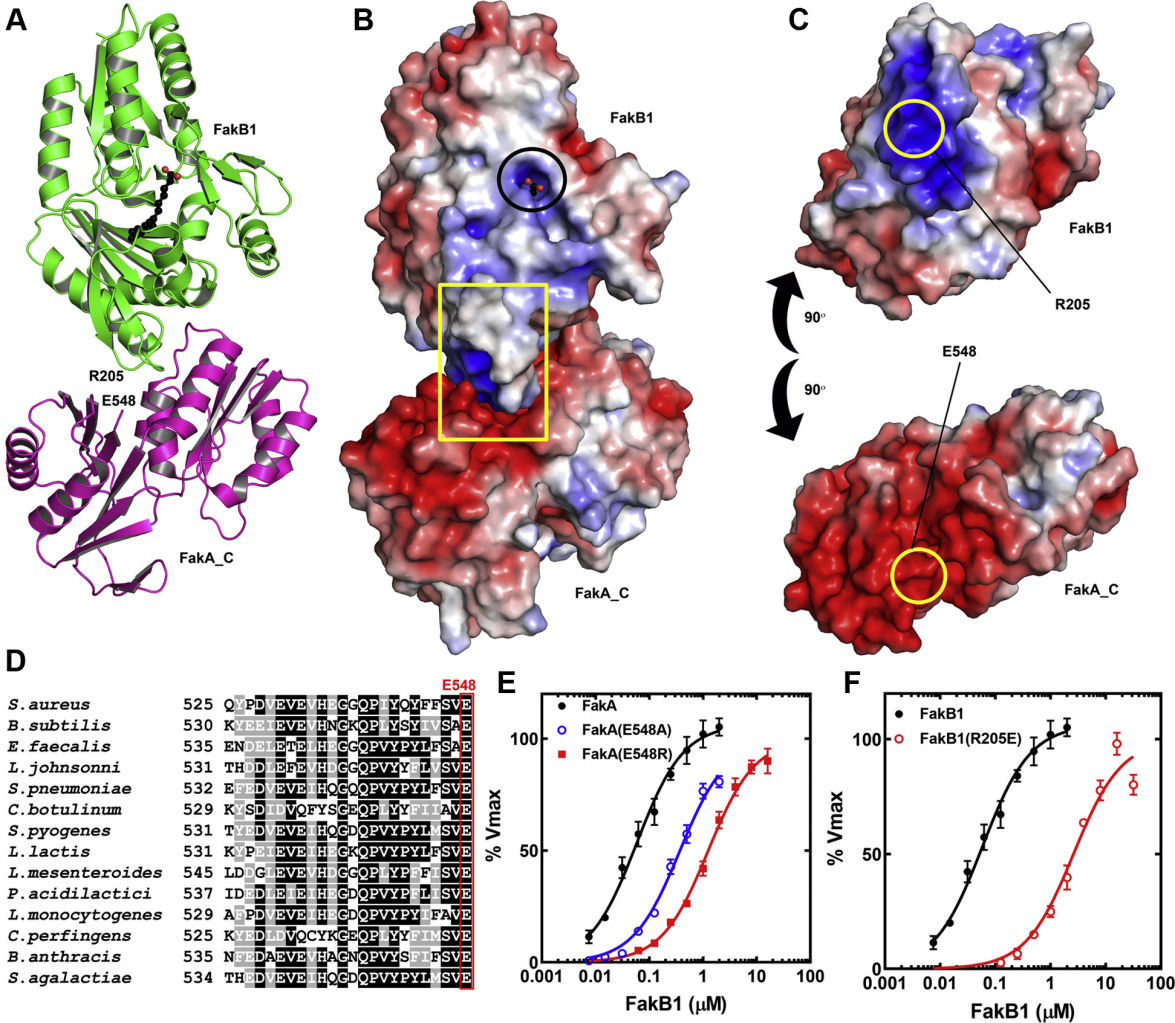
**FakA_C is the FakB substrate binding domain.***A*, docking model of FakB1 (*green*; PDBID: 5WOO) bound to FakA_C (*magenta*; PDBID: 6W6B) generated using HADDOCK 2.4. *B*, space filling electrostatic surface charge distribution of the modeled FakA_C•FakB1 complex (*blue*, positive; *red*, negative). The exposed carboxyl of the bound FA phosphorylated by FakA is *circled* and the *yellow rectangle* locates the FakB1•FakA_C interface. *C*, rotation of FakA_C and FakB1 90° showing the match between the negatively charged (*blue*) wedge on the surface of FakB1 and the positively charged (*red*) groove on FakA_C. The locations of the interacting Arg205 and Glu548 are *circled*. *D*, pile-up of 14 Firmicute FakA sequences showing the conservation of the C-terminal Glu548. *E*, FakB1 K_M_ for FakA (0.09 ± 0.02 μM; n = 10), FakA(E548A) (0.37 ± 0.04 μM; n = 4), and FakA(E548R) (1.25 ± 0.12 μM; n = 4). *F*, apparent K_M_ of FakA for FakB1 (panel *E*) compared to FakB1(R205E) (2.6 ± 0.4 μM; n = 4). The K_M_ values were calculated using nonlinear Michaelis–Menten regression analysis using GraphPad software (mean ± SE). FakA, kinase component of fatty acid kinase; FakB, fatty acid binding protein component of fatty acid kinase.

Because FakA_C binds FakB1 (Fig. 4*C*), we docked FakB1 and FakA_C using HADDOCK (24, 25) to gain insight into the residues involved in this interaction. The highest scoring solution had a calculated binding energy of −599.3 kcal/mol showing that complementary surfaces in terms of shape and charge mediate the interaction between FakB1 and FakA_C (Fig. 9*A*). The space filling electrostatic surface charge distribution of the modeled FakA_C•FakB1 complex shows the 1375 Å^2^ of buried surface in the proposed complex (Fig. 9*B*). A 90° opposite rotation of the two proteins shows that a positively charged wedge on FakB1 centered on Arg205 binds to a negatively charged groove on FakA_C centered on Glu548 (Fig. 9*C*). A pile-up of FakA sequences shows that Glu548 in particular, and the negatively charged surface in general, are conserved features of the protein in Firmicutes (Fig. 9*D*). Two FakA mutants were prepared to test the role of Glu548. FakA(R548A) and FakA(E548R) both exhibited higher FakB1 K_M_ values (Fig. 9*E*) consistent with the requirement for a negatively charged residue in this position for optimal FakB binding. The converse experiment comparing the apparent FakB1 K_M_ to that of FakB1(R205E) shows that the affinity of FakB1(R205E) for FakA is reduced (Fig. 9*F*). These biochemical results corroborate the docking model for FakA_C and FakB1. Interestingly, the positively charged FakB1 wedge centered on Arg205 is the feature responsible for FakB1 binding to phospholipid bilayers to allow FA exchange at the membrane (12).

### Solution structure of FakA

**Figure 10:**
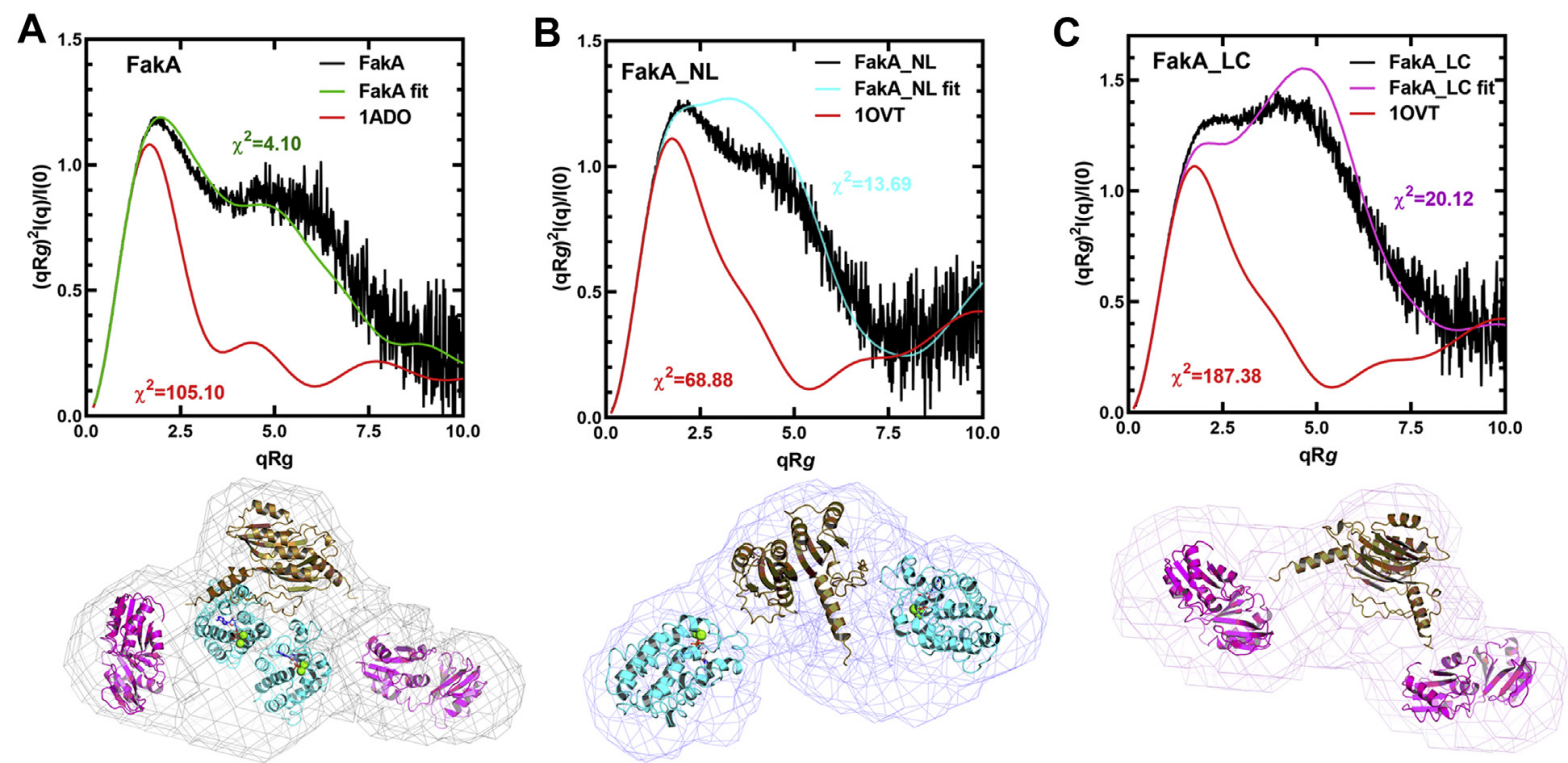
**Analysis of the FakA solution dynamics by SEC-SAXS.***A*, the dimensionless Kratky plots of FakA SAXS data (*black*) compared to the theoretical Kratky plot for a globular protein the size of FakA (aldolase; *red*) (PDBID: 1ADO) and to the theoretical Kratky plot (*green*) calculated from a model of the FakA domains fit into the SAXS volume as shown below. *B*, the dimensionless Kratky plots of FakA_NL SAXS data (*black*) compared to the theoretical Kratky plot for a globular protein the size of FakA_NL (ovotransferrin; *red*) (PDBID: 1OVT) and to the theoretical Kratky plot (*cyan*) calculated from a model of the FakA_NL domains fit into the SAXS volume as shown below. *C*, the dimensionless Kratky plots of FakA_LC SAXS data (*black*) compared to the theoretical Kratky plot for a globular protein the size of FakA_LC (ovotransferrin; *red*) (PDBID: 1OVT) and to the theoretical Kratky plot (*magenta*) calculated from the model of the FakA_LC domains fit into the SAXS volume as shown below. The χ^2^ values reflecting the fit between the two theoretical models and the SAXS data are shown in each panel. FakA, kinase component of fatty acid kinase; FakB, fatty acid binding protein component of fatty acid kinase; SEC-SAXS, size-exclusion chromatography coupled with small angle X-ray scattering.

The shape and assembly of the FakA dimer in solution were investigated using size-exclusion chromatography coupled with small angle X-ray scattering (SEC-SAXS). Data were collected with three constructs containing the FakA_L dimerization domain: FakA, FakA_NL, and FakA_LC (Fig. 10). All three proteins behave as monodisperse dimeric particles based on theoretical values calculated from the sequence (Table S5). The Guinier plots for all three proteins were linear, indicating that there was no significant radiation damage during the exposure period (Fig. S5, *A*–*C*). The asymmetrical bell-shaped distance distribution P(r) functions show that all three proteins have elongated overall shapes, and the shoulders on the curves indicate the presence of flexible domains with cross-sections of ∼30 to 50 Å (Fig. S5, *D*–*F*). The maximum diameters of FakA (162 Å), FakA_NL (164 Å), and FakA_LC (191 Å) are larger than predicted for globular proteins.

The analysis of SAXS data using Kratky plots provide insights into the globularity and flexibility of proteins (26). A comparison of the FakA SAXS experimental data with the profile of a globular protein of about the same size shows that FakA is clearly not globular (Fig. 10*A*). Electron density maps were generated from the solution scattering data, and the FakA domain structures were fit into the lobes of the SAXS volume. A theoretical Kratky plot was then generated for the molecular organization of FakA domains depicted in Figure 10*A*. This model is a close fit to the experimental data (Fig. 10*A*). These calculations are consistent with the FakA assembly consisting of freely rotating ordered domains tethered to the dimerization domain. The Kratky plots for FakA_NL (Fig. 10*B*) and FakA_LC (Fig. 10*C*) also show these proteins have flexible domains. The *ab initio* electron density maps generated from the solution scattering data both resemble boomerangs and were similarly analyzed by placing the FakA domain structures into the lobes of the SAXS volume (Fig. 10, *B* and *C*). The experimental SAXS data are not consistent with a globular configuration in both FakA_NL and FakA_LC but is a closer fit to theoretical structures consisting of peripheral globular domains (FakA_N and FakA_C) linked to the FakA_L dimerization domain by flexible tethers (Fig. 10, *B* and *C*). These data reflect the requirement for the large movements that must occur in the FakA_N and FakA_C domains to accommodate the binding and release of the large FakB substrate.

## Discussion

**Figure 11:**
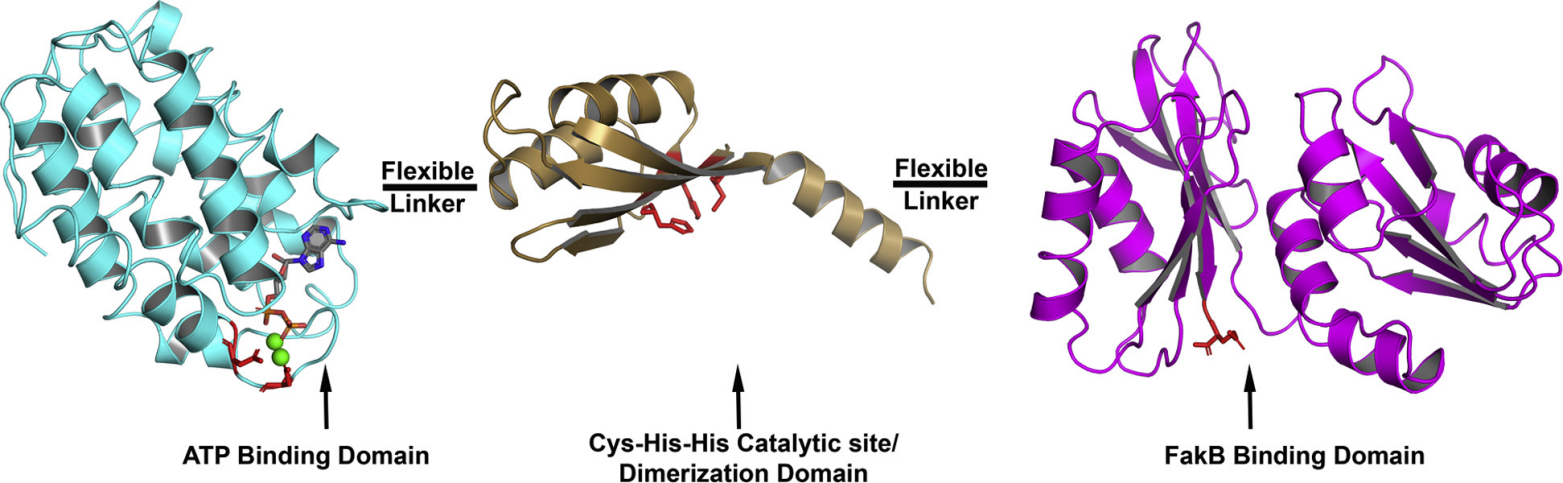
**Domain organization of FakA.** The FakA_L catalytic/dimerization domain (*chocolate*) is attached to the ATP-binding N terminus (FakA_N; *cyan*) and the FakB substrate-binding C terminus (FakA_C; *magenta*) with the dimer arranged in an antiparallel fashion. FakA_N and FakA_C are attached to FakA_L by 34 and 9 residue unstructured, flexible linkers, respectively, that allow the relative movement of the domains that is required to bind the large FakB substrate. FakA, kinase component of fatty acid kinase; FakB, fatty acid binding protein component of fatty acid kinase.

The FakA component of Fak consists of three independently folded domains, each with a specific role in catalysis (Fig. 11). The N-terminal domain, FakA_N, is highly related to DhaL of the Dha kinase family and tightly binds ADP•(Mg^2+^)_2_. FakA_N alone is inactive as a kinase, and our inability to obtain a crystal with ATP suggests that the ADP form is the most stable bound nucleotide in the absence of other FakA protein domains. In two-Mg^2+^ kinases, the rate of product release is slow due to the stabilization of ADP in the active site by the two Mg^2+^ ions (27), and it appears that we have captured the product complex in our FakA_N•ADP•(Mg^2+^)_2_ structure. Because FakA catalyzes the transfer of the γ-phosphate of ATP to the FA (7), the exchange of ADP for ATP must be facilitated by the interaction of FakA_N with other FakA domains during the catalytic cycle. FakA_C is the FakB binding component that presents the FA bound to FakB to the FakA active site. FakA_L contains the dimerization interface that we have localized to the αβ bundle–helix region of FakA_L. The AlphaFold prediction of an antiparallel orientation of the protomers is consistent with the SAXS analyses. FakA_L also contains the Cys-His-His triad that is required for catalysis that must close over the FakA_N•FakB•FakA_C complex to create the active site and execute catalysis. The lack of a structure showing the formed active site makes it difficult to assign specific functions to these residues, except to note that their molecular properties suggest that they are involved in neutralizing the negative charge on the ATP γ-phosphate to facilitate catalysis. Following the phosphotransfer reaction, the complex dissociates with the release of FakB•acyl-PO_4_. SAXS analysis leads to a model where the FakA_N and FakA_C globular domains are attached by unstructured linkers to the dimerization domain and move independently of each other in solution. The free movement of these components allows FakA to open and close around the FakB substrate, which is larger than either FakA_N or FakA_C. Finally, FakB also has a required role in forming the catalytic center. Arg173 of FakB1 is conserved in all FakBs but does not directly interact with the FA carboxyl. Rather, it is located above the FA carboxyl in a position to neutralize the negative charge on the FakB•acyl-PO_4_ product (9, 11, 28). The FakB(R173A) mutant is a stable protein that carries out FA exchange normally but is inactive in the Fak assay (11). This means that Arg173 on FakB, the Cys-His-His triad on FakA_L, and the nucleotide binding FakA_N all contribute to forming the active site of Fak. Defining the specific roles for these residues in catalysis will require further structural and biochemical experiments.

## Experimental procedures

### Materials

[1-^14^C]Palmitic acid (56.7 mCi/mmol) was from PerkinElmer Life Sciences. Antibiotics, high density nickel resin, and isopropyl β-d-thiogalactopyranoside were from GoldBio. All other reagents were from Sigma unless otherwise indicated.

### Trypsin digestion of FakA

The amount of time and trypsin concentration required to provide a partial digest of 1 mg/ml FakA in 20 mM Tris, pH, 7.5, 200 mM NaCl with or without 5 mM AMP, and 10 mM MgCl_2_ was empirically optimized using SDS gel electrophoresis to assess FakA digestion and product formation. The reactions were stopped by adding SDS loading buffer containing 1 μl of protease inhibitor cocktail. The presence of the nucleotide did not influence the results. The scale-up reaction used 5 mg/ml of FakA that was digested with 1 μg/ml of trypsin for 24 h at 24 °C. FakA was cut into five bands on SDS gels (Fig. S1*A*). Triplicate samples of the digest were analyzed by liquid chromatography time-of-flight mass spectrometry using a reverse phase column and Micromass Q-TOF mass spectrometer as described (29). Mass spectroscopy data were deconvoluted to calculate the molecular weights of the separated products and identify the two trypsin hypersensitive cut sites (Fig. S1*B*).

### Cloning and expression of FakA domains

The DNA for FakA_N, (1–624 bp) and FakA_LC (640–1647 bp) was ordered from Invitrogen and cloned into NdeI and XhoI sites in pET28b to obtain pKM327 and pKM326, respectively. FakA_L (601–982 bp), and FakA_C (982–1647 bp) were PCR amplified using pJLB11 as template and cloned into NdeI and XhoI sites in pET28b to obtain pRZ102 and pRZ103, respectively. FakA_NL (1–982 bp) was made by mutagenesis to change the M328 to a STOP (TAG) with pJLB11 as the template to obtain pPJ598. FakA_αβ-Bundle (745–981 bp) was constructed by Gibson Assembly of a PCR product of pJLB11 into NdeI and HindIII in pET28a to obtain pPJ600. FakA_αβ-bundle with loop (601–924 bp) and FakA_αβ bundle with helix (712–981 bp) were amplified from by PCR with strain AH1263 genomic DNA as a template and cloned into NdeI and XhoI in pET28a by Gibson Assembly cloning kit (NEB) to obtain pPJ633 and pPJ634, respectively. All the mutants of FakA were constructed by site-directed mutagenesis using QuickChange Lightning Site-Directed Mutagenesis Kit (Agilent) and pJLB11 as template. All proteins in the pET28 vector had an N-terminal His tag. The proteins were expressed in BL21(DE3) cells and purified by Ni^2+^ affinity chromatography. The list of plasmids is given in Table S6 and primers is given in Table S7.

### Stability and SEC

The proteins were dialyzed in 20 mM Tris, pH 7.5, 200 mM NaCl and were subjected to analytical size exclusion chromatography on a S-75 13/300 column and eluted with 20 mM Tris, pH 7.5, 200 mM NaCl. Protein standards were run on the same column to generate a standard curve for molecular weight *versus* elution volume to estimate the oligomerization state of the proteins. The proteins were also analyzed by thermal denaturation with SYPRO orange dye as described in (30). Briefly, 20 μM of each domain was mixed with a 1:200 dilution of SYPRO orange and subjected to thermal denaturation from 26 to 95 °C at a rate of 1 °C/min. Fluorescence was measured using a TAMRA filter set (Ex 560 nm and Em 582 nm) in ABI 7500 Fast thermal cycler. When present, ATP and MgCl_2_ are present at 10 mM. The data were plotted as fluorescence intensity *versus* temperature and were fit to the Boltzmann sigmoidal equation using GraphPad software to determine the temperature corresponding to the denaturation of 50% of the protein. Each experiment was performed in triplicate.

### Analytical ultracentrifugation

Experiments were conducted in a ProteomeLab XL-I analytical ultracentrifuge (Beckman Coulter) following standard protocols (31). All samples in a buffer containing 20 mM Tris, pH 7.5, 200 mM NaCl, and 5 mM DTT were loaded into a cell assembly composed of a double sector charcoal-filled centerpiece with a 12-mm path length and either quartz or sapphire windows. The density and viscosity of the ultracentrifugation buffer at 20 °C were measured with a DMA 5000 M density meter and an AMVn viscometer (both Anton Paar), respectively. The cell assembly in the sedimentation velocity experiments contained identical sample and reference buffer volumes of 300 μl was placed in a rotor and temperature equilibrated at rest at 20 °C for 2 h before it was accelerated from 0 to 50,000 rpm. Rayleigh interference optical data were collected at 1-min intervals for 12 h. The velocity data were modeled with diffusion-deconvoluted sedimentation coefficient distributions c(s) in SEDFIT (32), using algebraic noise decomposition and with signal-average frictional ratio and meniscus position refined with nonlinear regression. The s-value was corrected for time, temperature, and radial position, and finite acceleration of the rotor was accounted for in the evaluation of Lamm equation solutions (33).

### Fak assay

Fak assays were performed as described in (34). Briefly, the assay mixture contained 100 mM Tris HCl, pH 7.5, 20 mM MgCl_2_, 10 mM ATP, 0.1% Triton X-100, 20 μM [^14^C]palmitic acid (56.5 mCi/mol), and 1 μM FakB1 in a 60 μl assay volume. The assay was started by adding FakA or FakA domains or mutant FakA protein and performed at 37 °C for 15 min after which 45 μl was transferred to DE81 disks and washed three times with ethanol containing 1% acetic acid. Disks were dried and counted by scintillation counting. To determine the K_M_ for FakB1, the reaction mix containing FakA at 0.1 μM was added to FakB1 to start the reaction. For determining K_M_ for FakB1 and FakB1(R205A) with the FakA and FakA(E548R) [^14^C]palmitic acid (56.5 mCi/mol) was 50 μM and FakA was at 0.2 μM.

### Crystallization, data collection, and structure determination

Crystals of FakA_N were obtained at 291 K by the hanging drop method and were grown in two conditions. Form 1 was grown in 0.2 M MgCl_2_, 0.1 M Tris-HCl, pH 8.5, and 30% PEG4000, and form 2 was grown in 0.2 M ammonium formate, pH 7.5 and 20% PEG3350. Both forms are identical with the same space group (P2_1_2_1_2_1_) and unit cell parameters. X-ray diffraction data were collected on beam line 22-ID at the Southeast Regional Collaborative Access Team at the Advanced Photon Source, Argonne National Laboratory. All data were collected from crystals cryoprotected with 30% glycerol and frozen in liquid nitrogen, and the raw data were integrated using XDS (35) and scaled and merged using AIMLESS/CCP4 with (36) with 5% of the reflections flagged for calculation of the free R values. The initial structure was determined by single wavelength anomalous diffraction (SAD) phasing using a cobalt derivative. SAD data were collected to a resolution of 1.90 Å at the cobalt absorption K-edge of 1.6039 Å using crystal form 2 soaked for 16 h in 40 mM CoCl_2_. The mid slope of the anomalous normal probability is 1.358, reflecting the presence of a strong anomalous signal, and phasing was performed to a resolution of 2.4 Å based on the significance of the anomalous signal (Fig. S6*A*). The anomalous difference Patterson map clearly showed the presence of a single cobalt atom (Fig. S6*B*). The initial SAD structure was determined using SHELXC/SHELXD/SHELXE/HKL2MAP (37, 38) and incorporated native data collected from crystal form 1 at wavelength 1.00 Å to a resolution of 1.03 Å. The final structure was built using a combination of ARP/wARP (39, 40) and COOT (41), and PHENIX.REFINE (42) was used for the refinement and addition of water molecules. The initial refined structure showed a mixture of AMP and ADP at the active site, but data collected from crystallized (form 1) protein preincubated for 1 h at 20 °C in 250 mM AMPPNP (93%) showed only ADP at the active site. Data collection and refinement statistics for form 1 containing Mg^2+^ and ADP (PDBID: 7RM7), which is the structure referred to throughout the paper, are shown in Table 1. Two other structures were refined and deposited (Table S4); the initial structure of form 1 containing Mg^2+^ and a mixture of AMP and ADP (PDBID: 7SNB), and the form 2 derivative structure containing Mg^2+^, Co^2+^, and ADP (PDBID: 7RZK). The SAD data statistics are reported in Table S4. The Co^2+^ was located in the position of the distal Mg^2+^ (Mg2) in in the 7RM7 structure (Fig. 5*B*).

Crystals of FakA_C were obtained at 291K by the hanging drop method in space group P3_1_21. The final crystallization conditions were a 2:1 volume ratio of precipitant (1 M Li_2_SO_4_, 1.4 M (NH_4_)_2_SO_4_) and protein (60 mg/ml in 10 mM Tris, pH 7.5, 1 mM DTT and 200 mM NaCl). Prior to data collection, crystals were immersed in a 1:1 mixture of paraffin oil and Paratone N as cryoprotectant prior to freezing in liquid nitrogen. The domain contains ten sulfur carrying residues, nine methionines, and one cysteine, and this allowed us to determine the X-ray structure using sulfur SAD phasing (43). Native data were collected at 1.00 Å wavelength to a resolution of 1.41 Å. Sulfur SAD data were collected at 1.70 Å to maximize the sulfur anomalous signal and from eight crystals to achieve the necessary redundancy to maximize the accuracy of the anomalous signal. Data processing and structure determination used the same approach as described for FakA_N. BLEND/CCP4 (44) confirmed that the eight crystals were sufficiently isomorphous for the inclusion of all the datasets. The mid slope of the anomalous normal probability is 1.276, which confirmed the presence of a sufficient anomalous signal (Fig. S6*A*), and the anomalous difference Patterson map (Fig. S6*C*) showed clear peaks corresponding to the sulfur atoms. The initial structure was determined at 2.4 Å, which was the resolution limit of the usable anomalous signal (Fig. S6*A*), and the final refined structure was deposited in the PDB (PDBID: 6W6B). Data collection and refinement statistics are shown in Table 1, and the SAD data statistics are reported in Table S4. We used AlphaFold protein structure prediction software (22) to model the structure of FakA and the FakA_L dimerization interface. ZDOCK was used to determine the initial SaFakB1-SaFakA_C complex. A preliminary model showed excellent surface shape and charge complementarity as viewed using APBS/PyMOL. HADDOCK (24, 25) was then used to refine the preliminary model.

### SEC-SAXS

SEC-SAXS experiments were performed at the BioCAT beamline 18-ID-D at the Advanced Photon Source. Photons that scattered from the λ = 1.033 Å X-ray beam were recorded on the Pilatus3 X 1M detector at a sample-to-detector distance of 3.6 m, accessing a range of momentum transfer (q) from 0.0042 to 0.35 Å^−1^. A Superdex 200 10/300 gel filtration column was pre-equilibrated with 20 mM Tris, pH 7.5, 200 mM NaCl, and 1% glycerol. The column was loaded with 300 μl FakA (4 mg/ml), 300 μl FakA_NL (6.2 mg/ml), or 300 μl FakA_LC (6 mg/ml) and flowed at a rate of 0.5 to 0.6 ml/min that enabled removal of any potential aggregates before flowing the FakA constructs through a temperature-controlled quartz capillary (1.0 mm internal diameter) flow cell for X-ray exposure. SAXS was performed in-line with this SEC setup. SAXS exposures (0.5 s) of fractions from the SEC column were recorded every second and showed one peak of X-ray scattering corresponding to the elution volume of the protein. Buffer background was obtained from a baseline region of the chromatogram and subtracted from the averaged central portion of the FakA construct peaks to obtain the scattering profiles. Data were reduced, processed, and the overall parameters computed following standard procedures of the software package RAW (45), version 2.1.0. The zero-angle intensity I_0_, radius of gyration R*g*, and associated uncertainties for these parameters were obtained by weighted linear regression of ln(I(q)) *versus* q^2^ as shown in the Guinier plot. The particle dimension Dmax was determined from the pair distance distribution function P(r) with the program GNOM (46). Reconstructions (n = 20) were aligned, averaged, and refined in slow mode using DENSS (47) to calculate the electron density for the final three-dimensional volume.

Theoretical scattering profiles and qualities of fit (χ^2^) were obtained from the FoXS webserver (48) using the atomic coordinates of aldolase from rabbit muscle (PDBID: 1ADO) or hen ovotransferrin (conalbumin) (PDBID: 1OVT) and the experimental SAXS profiles. The theoretical dimensionless Kratky plots for aldolase or ovotransferrin were computed in RAW (45) using the theoretical scattering profiles generated by FoXS (48). The models for FakA, FakA_NL, and FakA_LC were generated by placing the crystal structures of FakA_N and FakA_C, and the AlphaFold prediction for FakA_L (Fig. 7*C*), in the SAXS volumes to generate the dimers. The dimers were assembled by using the theoretical FakA_L dimer to orient the FakA_N and FakA_C domains and rationally align their respective termini. The theoretical scattering profiles and qualities of fit for the models to the experimental FakA, FakA_NL, and FakA_LC SAXS profiles were obtained from the FoXS webserver (48), and the theoretical dimensionless Kratky plots were computed in RAW (45).

## Data availability

The structural coordinates have been deposited in the Protein Data Bank as PDBID: 7SNB, 7RZK, 7RM7, and 6W6B. All other study data are included in the article or supporting information.

## Supporting information

This article contains supporting information.

## Conflict of interest

The authors declare that they have no conflicts of interest with the contents of this article.
