# Supplementary material for: Domain architecture and catalysis of the Staphylococcus aureus fatty acid kinase
Source: J Biol Chem. 2022 Apr 29;298(6):101993. doi: 10.1016/j.jbc.2022.101993 (PMC9136124; doi:10.1016/j.jbc.2022.101993)
Supplement: Supplemental Figures S1–S6 and Tables S1–S7 [file mmc1.pdf]

Supplementary Materials

Domain architecture and mechanism of the *Staphylococcus aureus* fatty acid kinase

Chitra Subramanian, Maxime G. Cuypers, Christopher D. Radka, Stephen W. White and Charles O. Rock

<u>CONTENTS</u>

Figures S1-S5

Tables S1-S7

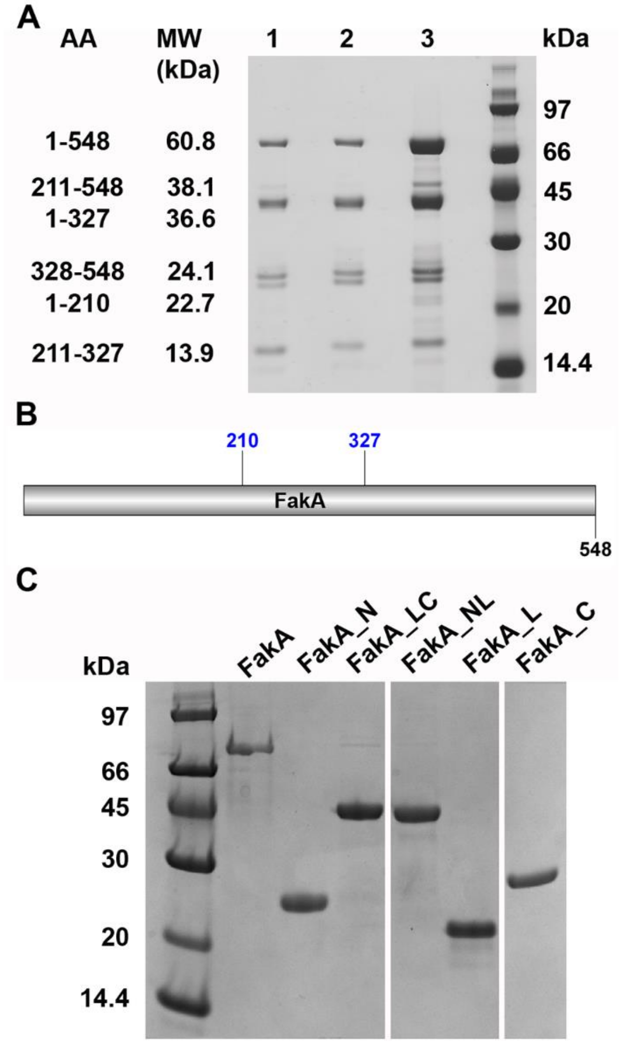

Figure S1. Determination of domains in FakA by limited trypsin digestion. *A*, Coomassie stained gel of trypsin digested FakA at RT for 24 h with 0.001 mg/ml trypsin: lane 1, 1 mg/ml FakA; lane 2, 1 mg/ml FakA plus 5 mM AMP plus 10 mM $MgCl_2$; lane 3, 5 mg/ml FakA plus 5 mM AMP plus 10 mM $MgCl_2$. The molecular weights and the amino acids contained in each fragment are given on the left. *B,* a schematic diagram showing the location of the trypsin sensitive lysines in FakA. *C,* Coomassie stained gel showing the purity of each domain purified using Ni^{2+}affinity chromatography. The image is from a single gel except that lanes 5, 8 and 9 containing proteins not used in this study are spliced out. The white lines indicate where the gel was cut.

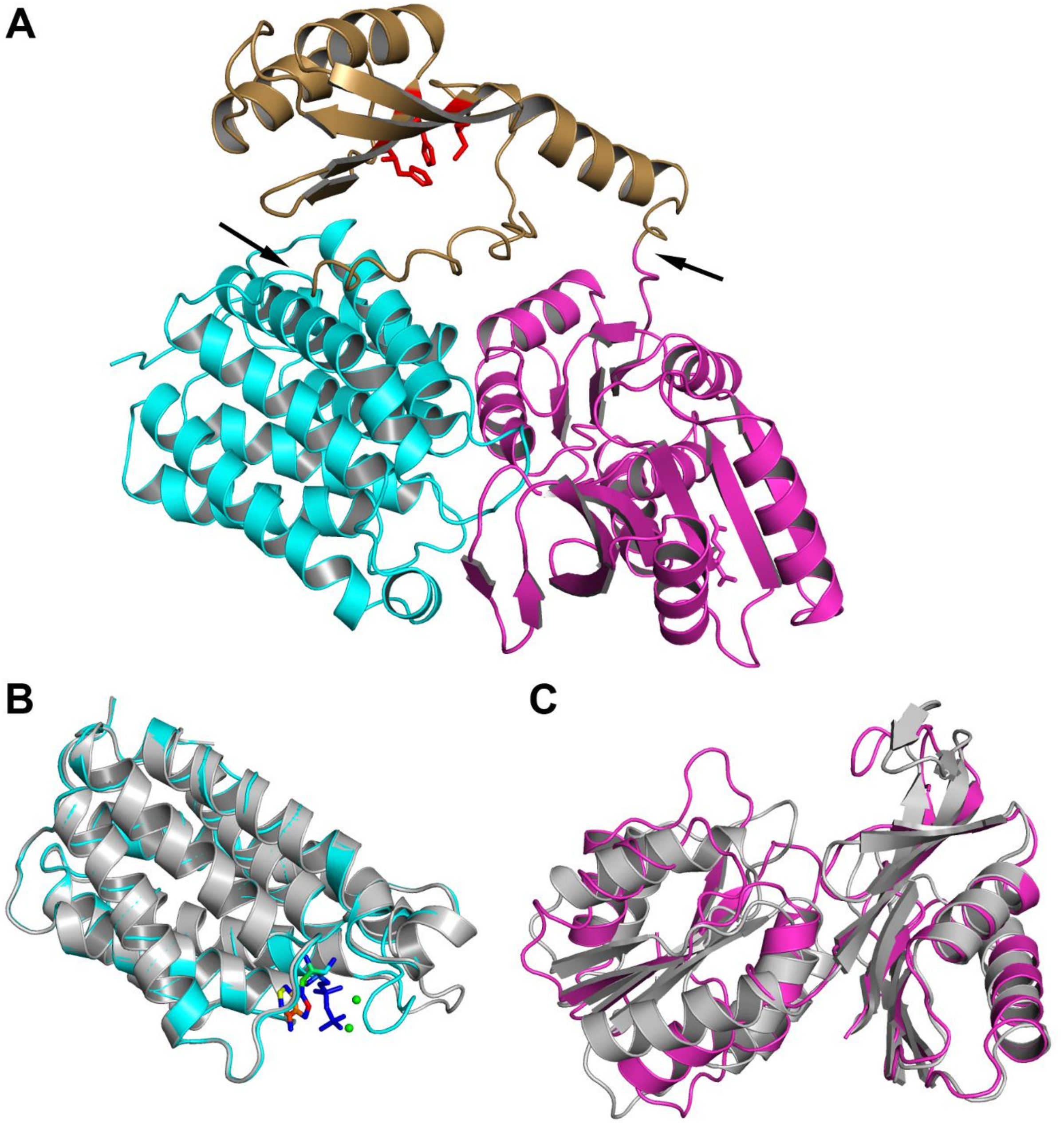

Figure S2. AlphaFold prediction of FakA. *A,* an AlphaFold prediction of a FakA protomer (accession number AF-Q2FZ58-F1). The locations of the proteolysis sites are indicated by arrows. The catalytic triad of Cys240, His282 and His284 are shown as red sticks. *B,* an alignment of the FakA_N AlphaFold prediction (grey) with the FakA_N crystal structure (cyan, PDBID: 7RM7) has an RMSD of 0.311 Å. *C*, an alignment of FakA_C AlphaFold prediction (grey) with the FakA_C structure (pink, PDBID: 6W6B) has an RMSD of 2.957 Å. Calculations were performed using SUPERPOSE/CCP4i.

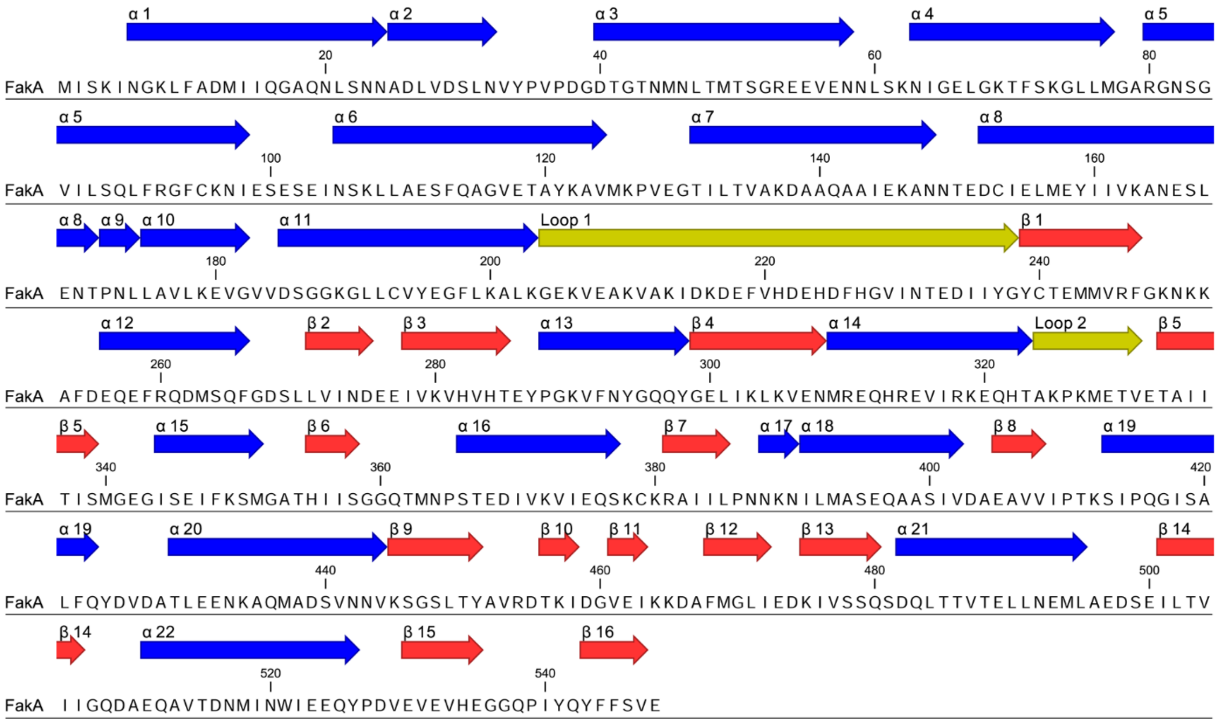

Figure S3. A consolidated secondary structure map of the α-helices and β-sheets of FakA. The α-helices are numbered α1-α22 (blue) and the β-sheets are numbered β1-β16 (red). The two unstructured loops (yellow) joining the dimerization domain to the N- and C-terminus are designated Loop 1 and Loop 2, respectively.

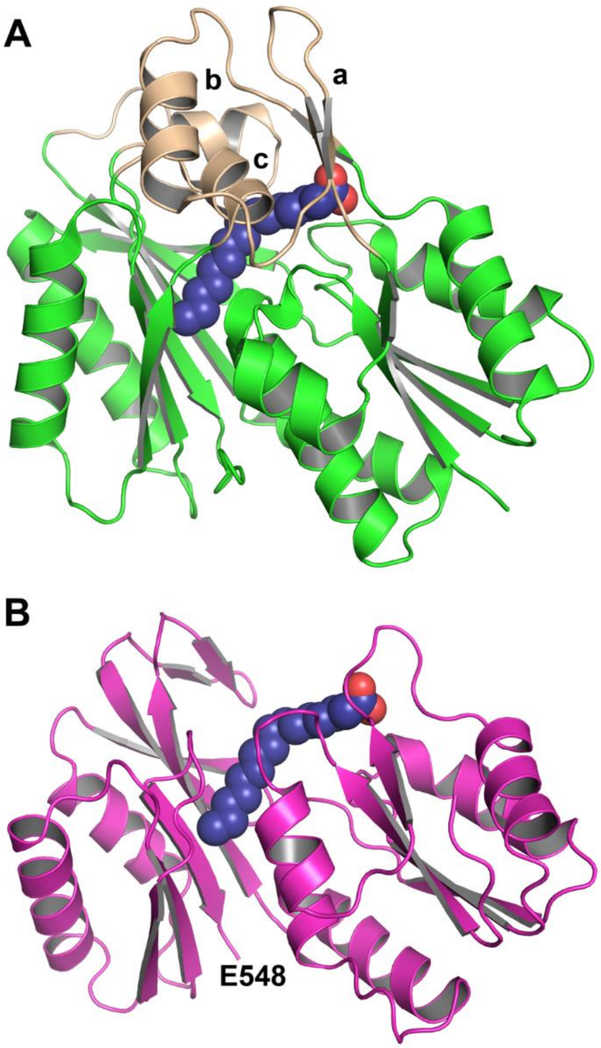

Figure S4. The FA tunnel in FakB1 is absent in FakA_C. *A*, a view of FakB1 (green) with the three structural elements (**a**, **b** and **c**) that are not found in FakA_C shown in tan (see Fig. 8). The FA is blue. The structural elements missing from the FakA_C primary sequence come together at a crevice at the subdomain interface to form the top of the FA acid binding tunnel. *B*, the FakA_C structure (magenta) with the FA (blue) placed where it would be located if the FakB1 tunnel existed in FakA_C. The absence of the insertion elements (tan) in FakA_C creates an open crevice on the FakA_C surface. The FakB binding site is centered on Glu548 on the opposite side of FakA_C relative to the crevice.

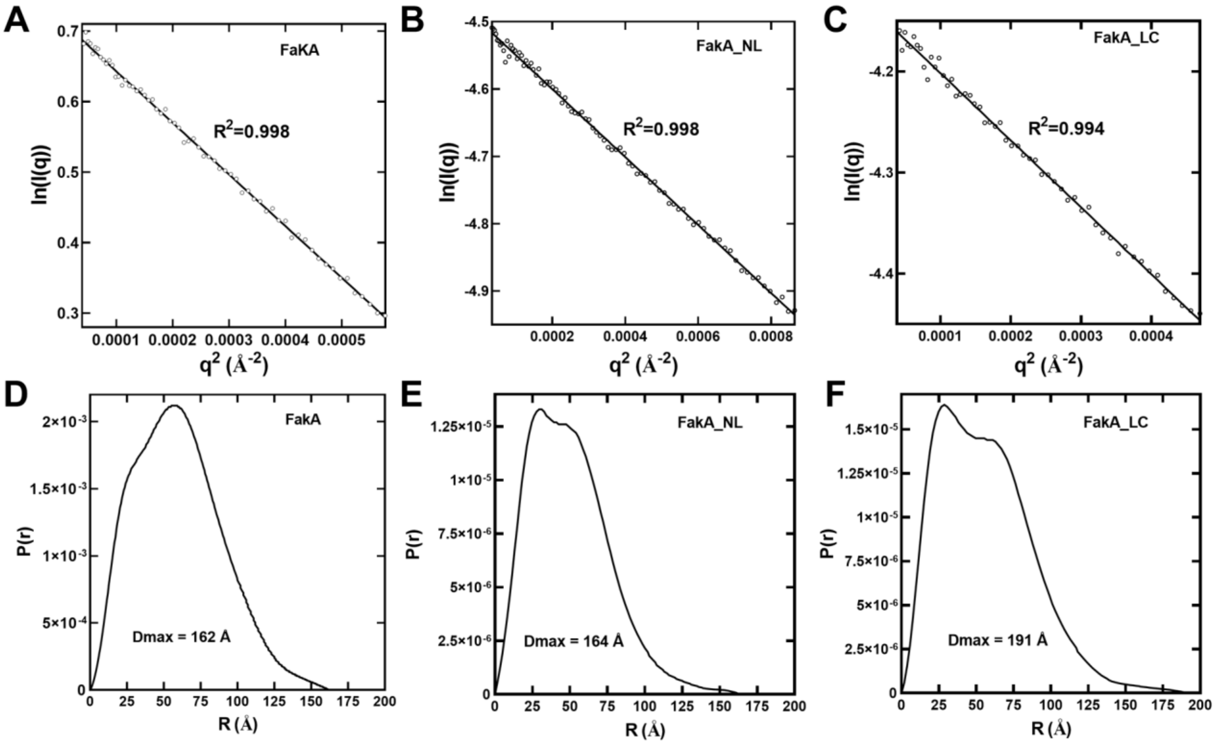

Figure S5. Guinier plot and Pr function of FakA, FakA_NL and FakA_LC. *A,* Guinier plot of FakA. *B,* Guinier plot of FakA_NL. *C,* Guinier plot of FakA_LC. The Linear Guinier plots indicated there was no significant radiation damage during the exposure period. *D-F*. Pr function of FakA, FakA_NL, FakA_LC.

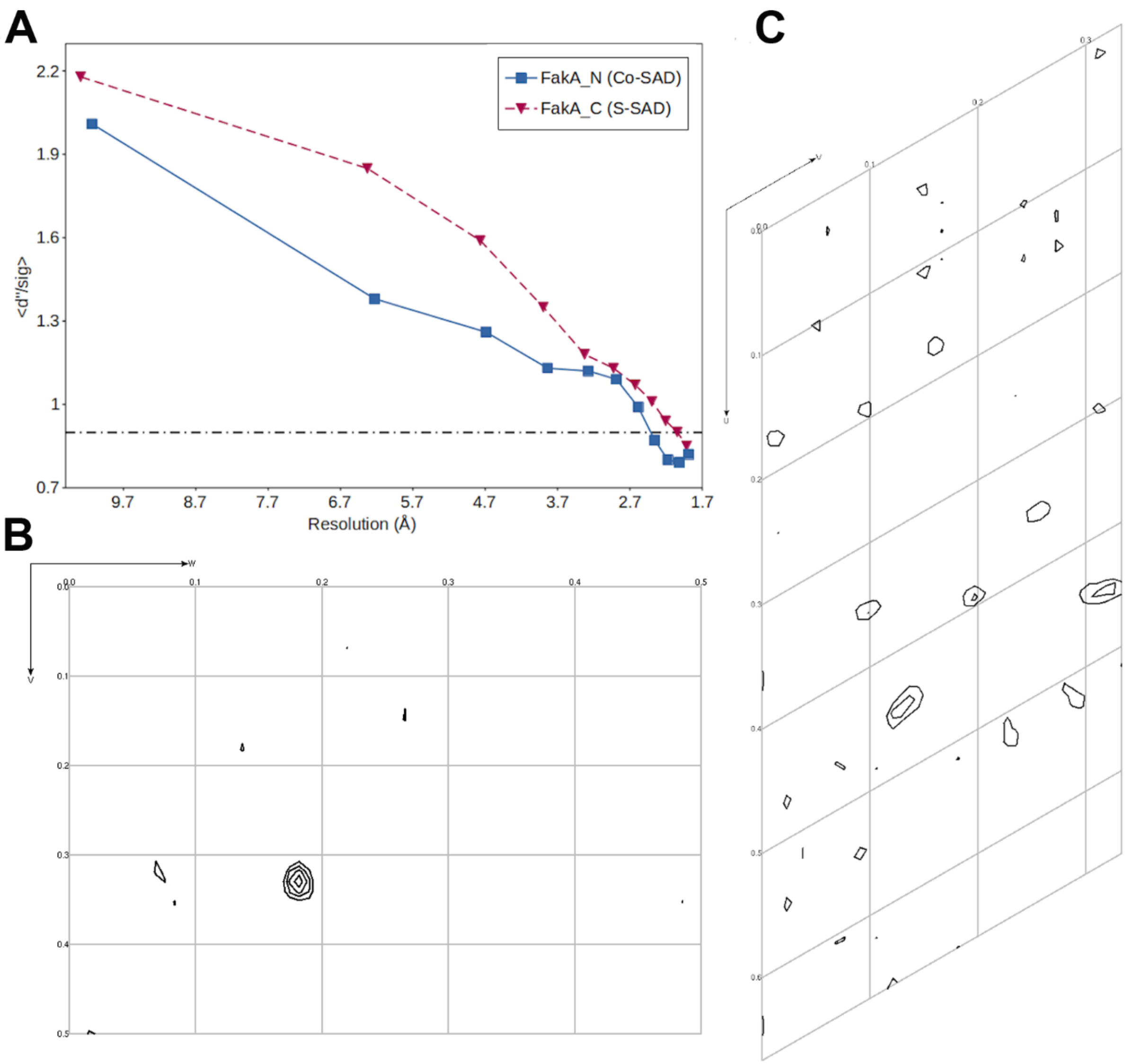

Figure. S6. The anomalous signal quality of the FakA_N (Co-SAD) and FakA_C (S-SAD) datasets. Patterson map computed using PATTERSON/CCP4i. *A*, the anomalous signal to noise ratio (<d”/sig>) for FakA_N (cyan) and FakA_C (magenta) calculated using SHELXC. *B*, FakA_N Harker section at X=0.5 (contour level = 2.5 to 10.0 sigma) showing the strong anomalous signal from Co^{2+}. *C*, FakA_C Harker section at Z=0.333 (contour level = 1.5 to 10.0 sigma) from 10 sulfur atoms.

Table S1. Molecular weights of the trypsin digested fragments dectected by LC-MS/MS

His-FakA (amino acids)	MW (kDa)	FakA (amino acids)
18-230	22735	1-210
18-347	36633	1-327
18-568	60797	1-548
231-568	38080	211-548
348-568	24182	328-548

Table S2. Sedimentation velocity c(s) analysis of FakA domains, FakB1 and the mixtures of FakA domains and FakB1

Sample	mg/ml[a]	s_{20} (Svedberg)[b]	Mw (kDa)[c]	$(f/f_0)w$[d]
FakA_N	2	2.18 (98%)	24.9 (24.4)	1.32
FakA_LC	1.92	4.02 (90%)	84.7 (40.1)	1.63
FakA_L	1.36	2.54 (74%)<br>3.75 (16%)	33.8 (17.3)<br>60.8	1.41
FakA_B1	2.03	2.72 (96%)	34.5 (34.2)	1.33
FakA_N + FakB1	4.23	2.24 (48%)<br>2.7 (52%)	26.4 (24.4)<br>35.1 (34.5)	1.34
FakA_L + FakB1	3.78	2.52 (90%)<br>3.22 (8%)	35.3 (34.5)<br>51 (17.3)	1.46
FakA_C	4.04	2.34 (94%)	28 (26.3)	1.33
FakA_C + FakB1	6.24	2.22 (36%)<br>3.48 (64%)	31.8 (26.3)<br>62.3 (60.8)	1.51
FakA_αβ Bundle	0.47	1.33 (32%)<br>2.08 (62%)<br>3.09 (6%)	11.6 (10.5)<br>22.8<br>41.2	1.38
FakA_Loop-αβ Bundle	0.15	1.78 (60%)<br>2.3 ((39%)	15.2 (14.7)<br>22.1	1.2
FakA_αβ Bundle-Helix	0.32	2.22 (98%)	21.9 (12.9)	1.27

[a] Concentration of peak in mg/ml.
[b] Sedimentation coefficient taken from the ordinate maximum of each peak in the best-fit c(s) distribution in 20 mM Tris pH 7.5, 0.2 M NaCl, 1 mM DTT buffer at 20 °C with percentage protein amount in parenthesis. Units are Svedberg (S).
[c] Molar mass values taken from the c(s) distribution that was transformed to the c(M) distribution. The theoretical monomer mass is given in parenthesis.
[d] Best-fit weight-average frictional ratio values $(f/f_0)w$ taken from the c(s) distribution.

Table S3. Thermal denaturation in the presence and absence of 10 mM ATP○Mg^{2+}

Protein	V50 (°C)	ΔT (°C)
FakA	37.5 ± 0.4	10
FakA + ATP	47.5 ± 0.04	
FakA_N	35 ± 0.2	2.8
FakA_N + ATP	37.8 ± 0.2	
FakA_LC	43.7 ± 0.3	0.5
FakA_LC + ATP	44.2 ± 0.2	
FakA_NL	37 ± 1.7	15.9
FakA_NL + ATP	52.9 ± 0.3	
FakA(C240A)	38.3 ± 0.2	9.4
FakA(C240A) + ATP	47.7 ± 0.5	
FakA(C240S)	38.9 ± 0.2	9.3
FakA(C240S) + ATP	48.2 ± 0.4	
FakA(E548A)	35.8 ± 0.4	13.1
FakA(E548A) + ATP	48.9 ± 0.1	
FakA(E548R)	34.6 ± 0.5	14
FakA(E548R) + ATP	48.6 ± 0.05	
FakA(H282A)	35.9 ± 0.4	12.4
FakA(H282A) + ATP	48.3 ± 0.07	
FakA(H284A)	36.4 ± 0.3	7.3
FakA(H284A) + ATP	43.7 ± 0.3	
FakA(D268A)	36.1 ± 0.6	11.9
FakA(D268A) + ATP	48. ± 0.3	
FakA(E242A)	32.7 ± 1.7	14.5
FakA(E242A) + ATP	47.2 ± 0.07	
FakB1(R205E)	53.8 ± 0.06	

Table S4. X-ray crystallography data collection statistics for SAD phasing.

**Protein Complex**	**FakA_N (SAD) ADP○Mg○Co**	**FakA_C (Sulfur-SAD)**
**PDB ID**	**7RZK**	**None**
**Data collection**		
Beamline	SER-CAT 22-ID	SER-CAT 22-ID
Temperature (K)	100	100
Wavelength (Å)	1.6039	1.7000
Space group	P212121	P3121
a, b, c (Å)	43.01, 57.52, 81.25	87.11 87.11 85.71
α, β, γ	90.00, 90.00, 90.00	90.00, 90.00, 120.00
Resolution range (Å)	81.25 – 1.90	85.71 – 1.92
Rsym or Rmerge	0.108 (0.868)	0.119 (0.411)
Rpim	0.033 (0.336)	0.009 (0.061)
Unique reflections	16525 (1041)	28974 (1647)
Redundancy	11.7 (7.5)	170.1 (38.7)
Mn I/σ(I)	12.6 (1.9)	57.3 (9.6)
CC (1/2)	0.998 (0.883)	1.000 (0.972)
Completeness (%)	100.0 (99.6)	98.9 (84.5)
Wilson B-factor (Å^{-2})	19.7	17.9
Anom. completeness	100.0 (100.0)	98.5 (78.6)
Anom. multiplicity	7.1 (7.1)	88.3 (20.6)
DelAnom CC(1/2)	0.252 (0.217)	0.375 (0.077)
Mid Slope Anom. P.	1.358	1.28
**Model quality**		
Rwork / Rfree	15.07 / 19.00	-
No. atoms		
Protein	1569	-
Ligand/ion	35	-
Water	179	-
B factor:		
All atoms	29.5	-
Protein atoms	27.9	-
Ligands	33.8	-
Solvent atoms	42.7	-
R.m.s. deviations:		
Bond lengths (Å)	0.02	-
Bond angles (°)	1.54	-
protein residues	208	-
Ramachandran plot:		
Favored (%)	98.5	-
Allowed (%)	1.5	-
Outliers (%)	0	-
Rotamer outliers (%)	0.6	-
Clashscore	2.5	-
Metal ions	Mg, Co	-

*Values in parentheses are for highest-resolution shell.

Table S5. Macromolecular properties of proteins from SEC-SAXS

Sample	MW[a]	MW[b]	R*g*[c]	R*g*[d]	R*g*[e]	Res[f]	Dmax[g]
FakA	62.7	138.2	46.84 ± 0.11	47.24 ± 0.08	51	50.1	162
FakA_NL	38.5	78.5	38.93 ± 0.08	40.12 ± 0.14	41.5	38.7	164
FakA_LC	40.1	88.3	44.61 ± 0.17	45.55 ± 0.15	46.9	37.1	191

[a] Theoretical monomeric molecular weight of the protein in kDa.
[b] Molecular weight in solution obtained by Bayes SAXS analysis in kDa.
[c] Radius of gyration ± standard deviation in Å derived from Guinier analysis.
[d] Radius of gyration ± standard deviation in Å derived from GNOM analysis.
[e] Radius of gyration ± standard deviation in Å derived from DENSS analysis.
[f] Fourier Shell Correlation Resolution in Å of the reconstruction from DENSS.
[g] Maximum particle dimension in Å derived from the pair distance distribution function.

Table S6. List of plasmids

Plasmid	Description	Source
pJLB11	pET28a FakA	[a]Parsons, et al.
pKM327	pET28b FakA_N	Present study
pKM326	pET28b FakA_LC	Present study
pPJ598	pET28b FakA_NL	Present study
pRZ102	pET28b FakA_L	Present study
pPJ600	pET28b FakA_αβ Bundle	Present study
pPJ633	pET28b FakA_Loop-αβ Bundle	Present study
pPJ634	pET28b FakA_αβ Bundle-Helix	Present study
pRZ103	pET28b FakA_C	Present study
pCS106	pET28A FakB1	[a]Parsons, et al.
pCS142	pET28a FakA(E548A)	Present study
pCS143	pET28a FakA(E548R)	Present study
pPJ647	pET28a FakA(C240A)	Present study
pPJ485	pET28a FakA(C240S)	Present study
pPJ641	pET28a FakA(E242A)	Present study
pPJ635	pET28a FakA(D268A)	Present study
pKM304	pET28a FakA(H282A)	Present study
pKM305	pET28a FakA(H284A)	Present study

[a] Parsons, J. B., Broussard, T. C., Bose, J. L., Rosch, J. W., Jackson, P., Subramanian, C., and Rock, C. O. (2014) Identification of a two-component fatty acid kinase responsible for host fatty acid incorporation by *Staphylococcus aureus*. *Proc. Natl. Acad. Sci. U. S. A.* **111**, 10532-10537

Table S7. List of primers

Primers	Sequence
FakA_NL M628STOP-F	GAACAGCACACAGCTAAACCGAAATAGGAAACGGTTGAAACA
FakA_NL M628STOP-R	TGTTTCAACCGTTTCCTATTTCGGTTTAGCTGTGTGCTGTTC
FakA_L 601 Nde1-F	GCTTCGCATATGGCGCTTAAAGGTGAAAAAGTTG
FakA_L 982Xho1-R	CTGCACTCGAGTTATTTCGGTTTAGCTGTGTGCTG
FakA_αβ-Bundle Nde1-F	GTGCCGCGCGGCAGCCATATGAAGAATAAAAAAGCCTTTGATG
FakA_αβ-Bundle H3-R	CGAGTGCGGCCGCAAGCTTCTATTTCGGTTTAGCTGTGTGC
FakA_C 982 Nde1-F	GCTCCCATATGGAAACGGTTGAAACAGC
FakA_C 1647 Xho1-R	CTCCGGCTCGAGTTATTCTACTGAAAAGAAATATTG
FakA_Loop- αβ-Bundle-F	GTGCCGCGCGGCAGCCATGCGCTTAAAGGTGAAAAAGTTG
FakA_Loop-αβ-Bundle-R	TGGTGGTGGTGGTGCTCGATTAATTTTCAACCTTAAGTTTAAT TAATTCACC
FakA_αβ-Bundle-helix-F	GTGCCGCGCGGCAGCCATGGCTATTGTACTGAAATGATGGTTC
FakA_αβ-Bundle-helix-R	TGGTGGTGGTGGTGCTCGATTATTTCGGTTTAGCTGTGTGC
